# Diagnostic potential of NRG1 in benign nerve sheath tumors and its influence on the PI3K-Akt signaling and tumor immunity

**DOI:** 10.1186/s13000-024-01438-9

**Published:** 2024-02-08

**Authors:** Suwei Yan, Jingnan Zhao, Pengyang Gao, Zhaoxu Li, Zhao Li, Xiaobing Liu, Pengfei Wang

**Affiliations:** Department of Neurosurgery, The Third Hospital of Hebei Medical University, No. 139, Ziqiang Road, Qiaoxi District, Shijiazhuang, 050051 Hebei Province P. R. China

**Keywords:** Benign nerve sheath tumor, NRG1, PI3K/AKT, M2 macrophage, Neutrophil, Biomarker, GEO database

## Abstract

**Objective:**

Benign nerve sheath tumors (BNSTs) present diagnostic challenges due to their heterogeneous nature. This study aimed to determine the significance of NRG1 as a novel diagnostic biomarker in BNST, emphasizing its involvement in the PI3K-Akt pathway and tumor immune regulation.

**Methods:**

Differential genes related to BNST were identified from the GEO database. Gene co-expression networks, protein-protein interaction networks, and LASSO regression were utilized to pinpoint key genes. The CIBERSORT algorithm assessed immune cell infiltration differences, and functional enrichment analyses explored BNST signaling pathways. Clinical samples helped establish PDX models, and in vitro cell lines to validate NRG1’s role via the PI3K-Akt pathway.

**Results:**

Nine hundred eighty-two genes were upregulated, and 375 downregulated in BNST samples. WGCNA revealed the brown module with the most significant difference. Top hub genes included NRG1, which was also determined as a pivotal gene in disease characterization. Immune infiltration showed significant variances in neutrophils and M2 macrophages, with NRG1 playing a central role. Functional analyses confirmed NRG1’s involvement in key pathways. Validation experiments using PDX models and cell lines further solidified NRG1’s role in BNST.

**Conclusion:**

NRG1 emerges as a potential diagnostic biomarker for BNST, influencing the PI3K-Akt pathway, and shaping the tumor immune microenvironment.

**Supplementary Information:**

The online version contains supplementary material available at 10.1186/s13000-024-01438-9.

## Introduction

A benign nerve sheath tumor (BNST) is a type of tumor that develops from the peripheral nerve sheath. It is frequently associated with neurofibromatosis and solitary neurofibroma [1]. Despite being a benign tumor, the surgical removal of it can be challenging due to its location, size, and its relationship with vital structures [2]. In certain cases, the complete removal of a tumor without causing harm to vital nerves or adjacent structures necessitates the use of highly sophisticated surgical techniques [3]. Damage to important nerves or structures during surgery can result in postoperative functional impairments or complications [4]. Although the BNST is considered benign, it is still important to acknowledge the possibility of local recurrence, necessitating close follow-up [5]. In conclusion, despite the benign nature of BNST, its clinical significance cannot be ignored due to the challenges associated with its treatment and the high rate of local recurrence [6].

Single-cell transcriptome sequencing offers a distinct method for comprehensively investigating the tumor microenvironment at the individual cell level [7]. This technology enables researchers to explore the heterogeneity and functional states of various immune cells within tumors [8]. This high-resolution perspective provides a comprehensive view of the intricate immune infiltration landscape within tumors, allowing for a deeper understanding of the mechanisms by which tumors evade immune surveillance [9]. Moreover, this method also aids in the identification of potential biomarkers that can forecast the progression of diseases or the response to treatment [10]. Hence, single-cell transcriptome sequencing has not only enhanced our comprehension of the tumor immune microenvironment, but has also furnished a potent instrument for tailored therapy [9]. The utilization of this technology signifies the advancement of more precise and targeted immunotherapy approaches, which can result in improved treatment outcomes and prognosis for patients [11].

This study thoroughly examined the molecular characteristics of the BNST using single-cell transcriptomic sequencing technology. By conducting a comprehensive analysis of gene expression in numerous individual cells, NRG1 was identified as a prospective molecular marker [12]. Previous literature suggests a strong correlation between NRG1 and the development and progression of neuro-related tumors [13]. Furthermore, it is also believed that NRG1 is associated with the immune microenvironment of the tumor, thereby impacting the infiltration and functionality of immune cells [14]. This novel finding establishes a connection between NRG1 and BNST, which offers a potential molecular marker for diagnosing BNST [15]. Further research should investigate the precise role of NRG1 in the pathogenesis of BNST and assess its potential implications for treatment and prognosis [16]. In conclusion, the discovery of NRG1 not only enhances our comprehension of BNST, but also offers a novel avenue for diagnosing and treating neurogenic tumors [15].

The objective of this study is to investigate the potential diagnostic biomarkers of the BNST and the corresponding underlying molecular mechanisms. Researchers have identified NRG1 as a novel diagnostic biomarker, indicating that it has a significant role in the development of BNST. Moreover, this study revealed that NRG1 potentially regulates M2 macrophages and neutrophils in the immune microenvironment by influencing the PI3K-Akt pathway, thereby contributing to the formation of BNST. These findings offer novel insights for developing therapeutic strategies targeting BNST.

## Materials and methods

### Transcriptome sequencing data from public databases

The Gene Expression Omnibus (GEO) database (http://www.ncbi.nlm.nih.gov/geo/) was queried to identify transcriptome RNA sequencing datasets associated with the BNST. Datasets GSE39645 and GSE54934, both containing standard control samples, were selected. Following the removal of batch effects, the two datasets were merged. Specifically, GSE39645 consisted of 9 standard control samples and 31 vestibular BNST tissues, while GSE54934 included 9 standard control samples and 31 vestibular BNST tissues. It is important to note that, since these data were obtained from publicly accessible databases, there was no requirement for ethical approval or informed consent.

### Differential gene expression analysis

The mRNA expression profile related to the BNST was retrieved from the GEO database (https://www.ncbi.nlm.nih.gov/geo/). To refine the dataset, microRNA, lncRNA, methylation, and duplicate data were excluded, ultimately leading to the selection of two datasets, namely GSE39645 and GSE54934. The removal of batch effects and dataset merging were accomplished using the “SVA” package in R. Subsequently, differential gene expression analysis was performed using the “limma” package, with filtering criteria established as |logFC| > 2 and *P* < 0.05. Visualization of the results involved generating a heatmap and a volcano plot using the “pheatmap” and “ggplot” packages, respectively. The entire analytical process was executed using R version 4.2.1, developed by the R Foundation for Statistical Computing.

### WGCNA analysis

Weighted Gene Co-expression Network Analysis (WGCNA) was performed utilizing the “WGCNA” package in R software. The analytical procedures comprised the following steps: Initially, the median absolute deviation (MAD) for each gene was determined, and the bottom 5% of genes with the smallest MAD were excluded. Subsequently, the “goodSamplesGenes” function was employed to refine the expression matrix of differentially expressed genes (DEGs), eliminating genes that did not meet the quality criteria, thus forming an unweighted co-expression network. Next, the optimal soft thresholding parameter, denoted as β, was selected through the “pickSoftThreshold” function, facilitating the transformation of the adjacency matrix. Subsequent calculations involved the derivation of the Topological Overlap Matrix (TOM), upon which a hierarchical clustering dendrogram was constructed to group genes with similar expression patterns into distinct modules. The minimum number of genes within each module was defined as the threshold for the clustering height, thereby facilitating the merging of potentially related modules. Finally, the expression profiles of each module were summarized using module eigengenes (ME), and the correlation between ME and specific traits was computed. Modules that exhibited the highest relevance to the traits of interest were subsequently selected for further analysis [17–19].

### Construction of protein-protein interaction networks (PPIs)

The STRING database encompasses a comprehensive range of information, including experimental data, text-mining findings extracted from PubMed abstracts, and data from various other databases. Moreover, it employs bioinformatics methodologies to forecast protein-protein interactions, rendering it an invaluable resource for investigating protein interactions. In our study, we conducted an analysis of protein interactions involving the differentially expressed genes (DEGs) extracted from the combined dataset by leveraging this database. We applied a confidence threshold of 0.7 and confined our analysis to interactions within the human species. Subsequently, we utilized Cytoscape 3.7.2 software to visualize and selectively identify the top ten hub genes from the resulting network.

### LASSO regression analysis is used to select disease-related genes

Least Absolute Shrinkage and Selection Operator (LASSO) regression possesses two distinctive attributes. Firstly, it is well-suited for fitting generalized linear models and serves as a valuable tool for both variable selection and complexity adjustment, effectively mitigating issues related to overfitting. In our analysis, we employed the glmnet package within the R environment to carry out LASSO regression. Within a given sample set, samples with an RS (Risk Score) value below the average Risk Score across all samples were categorized as low-risk, whereas those with RS values exceeding the average were classified as high-risk. Consequently, we were able to identify the candidate number of genes that minimized error along with the corresponding log(λ) value as part of our analysis [20].

### ROC curve analysis

Utilizing both the training and validation datasets, we harnessed the capabilities of the R language’s pROC package to generate ROC curves. These curves were constructed based on the expression values of candidate genes, allowing us to assess the accuracy of disease status prediction derived from gene expression data [21].

### Immunohistochemical analysis

CIBERSORT, an algorithm rooted in gene expression analysis, was harnessed to elucidate the cellular composition within intricate tissue samples. To scrutinize the immune cell makeup within neurofibromatosis tumor patient specimens, we leveraged the “e1071” and “preprocessor” packages in the R software, complemented by the CIBERSORT algorithm. The quantification of immune cell levels in each sample was executed, followed by 100 simulations to identify outcomes with a significance threshold of *p* < 0.05. Employing the “corrplot” package in the R software, we visually represented these computations, generating both bar charts and correlation plots for immune cell populations. Subsequently, a differential analysis of immune cells was conducted using the “vioplot” package in R, and correlation tests, specifically Spearman tests, were carried out using the “ggplot2,” “ggpubr,” and “ggExtra” packages. Results with a significance level of *p* < 0.05 were selected for further analysis [22, 23].

### Gene function enrichment analysis

Conducting GO and KEGG enrichment analyses on the dataset comprising differentially expressed genes was accomplished using the “clusterProfiler” package within the R programming language. Subsequently, visual representations in the form of bar plots were generated to depict the outcomes of enrichment analyses across three categories: biological processes (BP), cellular components (CC), and molecular functions (MF) in the Gene Ontology framework. Additionally, bar plots were crafted to illustrate the results of KEGG pathway enrichment analyses, with the aid of the “enrichplot” package [24].

### scRNA-seq data analysis

The scRNA-seq data analysis was conducted employing the R package “Seurat” (version: v4.1.1). Initial quality control measures were implemented, with filtering criteria established as follows: nFeature_RNA > 500, nCount_RNA > 1000, nCount_RNA < 20,000, and percent.mt < 10. Subsequently, batch effects were mitigated through Canonical Correlation Analysis (CCA). Standardization procedures and principal component analysis (PCA) were executed using the LogNormalize function. The pivotal principal components essential for TSNE clustering analysis were selected. Marker genes for individual cell clusters were identified using the SingleR package, and cell annotation was carried out using the MouseRNAseqData function to access the reference dataset. Furthermore, pseudotime analysis was performed using the “monocle” package, while DDRTree was utilized for dimensionality reduction data analysis. Lastly, cells were sorted based on the expression trends of specific genes, and the trajectory construction was completed [25, 26].

### TSNE cluster analysis

To diminish the dimensionality of the scRNA-Seq dataset, an initial Principal Component Analysis (PCA) was conducted, focusing on the top 2000 genes displaying high variability in expression. Subsequently, employing the Elbowplot function within the Seurat package, the selection was narrowed down to the first 20 principal components for further analysis. To identify significant cell subpopulations, the FindClusters function from Seurat was employed, utilizing the default resolution value (res = 1). Subsequently, the t-SNE algorithm was applied to carry out non-linear dimensionality reduction on the scRNA-seq sequencing data, with the Seurat package aiding in the identification of marker genes for distinct cell subtypes. Finally, the cell annotations were made utilizing recognized lineage-specific marker genes and integrated with online databases for comprehensive analysis [27].

### Sample collection

During the tumor resection surgeries of five sporadic BNST patients, tissue samples were systematically collected and expeditiously preserved in Eagle’s medium with high glucose (DMEM) sourced from Gibco (#11,965,092, New York, USA). It is noteworthy that all samples and associated records were meticulously anonymized before the initiation of the research, and they were acquired prior to any radiotherapy or adjuvant treatments. Furthermore, we also procured three tissue samples from the vestibular nerve to serve as experimental controls, constituting the control group [28]. The study was conducted in full compliance with ethical standards, with prior informed consent forms being duly obtained from all patients and their respective family members. Moreover, this research endeavor was approved by Ethics Committee of The Third Hospital of Hebei Medical University. In addition to facilitating comprehensive pathological assessments, these invaluable samples were instrumental in the creation of Patient-Derived Xenograft (PDX) models and the establishment of distinct cell lines [29].

### Construction of the BNST-PDX model and inhibitor treatment

All animal experiments conducted in this study were approved by the Animal Ethics Committee at The Third Hospital of Hebei Medical University. Clinical tumor samples were meticulously sectioned into 1 mm fragments, and subsequently, they were transferred into a culture medium comprising high-glucose DMEM (Gibco, #11,965,092), supplemented with 156 U/ml of Type I collagenase (Gibco, #17,018,029), 1.25 U/ml of dispase grade I (Gibco, #17,105,041), 10,000 IU of P/S (Gibco, #15,140,163), and 50 mg/ml of gentamicin. The samples were subjected to an incubation period lasting 16–18 h. Following the incubation, the cells were subjected to a washing step involving 30 ml of high-glucose DMEM medium containing 10% fetal bovine serum (FBS of Australian origin, Gibco, #10,100,147), utilizing centrifugation at 800 rpm for 5 min. Subsequently, the cells were resuspended in 1 ml of advanced DMEM/F12 basal medium (Gibco, #12,634,010) containing 10% FBS, 1% P/S, 4 μm forskolin (Gibco, #344,270–25), 10 ng/ml β-herregulin (BD Biosciences, #396HB-050, New Market, MD, USA), and 2.5 µl/ml insulin (Sigma, #I6634). For the in vivo experiments, a Hamilton syringe (Sigma-Aldrich, 22,192-U) was employed to slowly inject 3 µl of the cell suspension, encompassing 10^6 cells (PDX-NC, PDX-shNrg1-1, PDX-shNrg1-2), into the sciatic nerve sheath of 6-week-old NOD/SCID male mice (Strain NO.T001492, GemPharmatech). This injection process was executed within a brief duration of 45–60 s to prevent any unintended leakage [29, 30].

The commencement of treatment was initiated once the average tumor diameter reached 0.3 mm [31]. PI3K inhibitors, namely BEZ235 (MedChemExpress, HY-50,673) and AZD8055 (MedChemExpress, HY-10,422), were meticulously dissolved in a mixture comprising 10% DMSO and 90% corn oil (MedChemExpress, HY-Y1888) until a final concentration of 30% was achieved. For the treatment regimen, PDX mice were subjected to daily oral gavage of BEZ235 at a dosage of 20 mg/kg, and this was administered continuously for 14 days [32]. Additionally, AZD8055 was administered via oral gavage at a volume of 0.1 ml/10 g body weight, twice weekly [33]. To activate the PI3K/AKT signaling pathway, a daily intracerebroventricular injection of the PI3K allosteric activator 740 P-Y, at a dosage of 10 mg/kg, was administered to PDX mice that had been established with sh-Nrg1 [34]. The mice were humanely euthanized, and tissue samples were collected once the transplanted tumors had reached a volume ranging from 800 to 1000 mm^3. The specific groups for inhibitor treatment encompassed PDX-con, PDX-BEZ235, and PDX-AZD8055, each comprising three mice. As for the activator-treated specific groups, they included sh-Nrg1 and sh-Nrg1 + 740 P-Y, each consisting of three mice [31–34].

### Construction of BNST cell lines and inhibitor treatment

The xenograft (P1) was dissociated into individual cells, which were subsequently cultured in DMEM medium at 37 °C with 5% CO2. The culture medium consisted of 10% FBS and 1% P/S. Passaging was performed every 3–4 days, and during this process, cells were evenly preserved by freezing them in a medium containing 10% DMSO, either in liquid nitrogen or at -80 °C for future use [29]. It is noteworthy that cells within 5 generations were exclusively utilized to prevent aging. The PI3K inhibitors, BEZ235 (MedChemExpress, HY-50,673) and AZD8055 (MedChemExpress, HY-10,422), were dissolved in DMSO. Subsequently, the cells were uniformly seeded in a 24-well plate at a density of 5 × 104 cells per well, and they were cultured under suitable conditions with the addition of growth medium. The final concentration of BEZ235 was set at 100 nM, and cells were harvested after 72 h of treatment [28]. For the AZD8055 treatment group, the final concentration was 50 nM, and cells were collected after 48 h of treatment [29]. Protein extraction was performed for subsequent analyses. The specific experimental groups encompassed the control group (con group), the BEZ235 group, and the AZD8055 group.

### Recombinant vector construction and cell infection

The vector pGMLV-SC5, obtained from BioVector NTCC Inc, was subjected to linearization through the action of double restriction enzymes, namely BamHI (FD0054, Thermo Fisher Scientific) and EcoRI (FD0274, Thermo Fisher Scientific). Subsequently, the linearized vector was purified using the Gel-Spin DNA Extraction Kit (DP204, Tiangen Biotech). To facilitate the connection of two short-hairpin RNAs (shNRG1-1 and shNRG1-2) designed to target NRG1, or a negative control shRNA (shNC), the enzymatic action of T4 Ligase (EL0011, Thermo Fisher Scientific) was employed. The specific sequence information can be found in Table S1.

The constructed vector was mixed with DH5α competent cells (CB101-02, Tiangen Biotech) and subsequently transfected into 293T cells (China Type Culture Collection, Chinese Academy of Sciences) following purification. The concentrated lentiviral solution, characterized by a high titer (> 108 TU/ml), was harvested from the clarified supernatant. The shRNA was diluted to a concentration of 0.1 nmol for transfection purposes. The constructed cell lines were infected with the lentivirus at an MOI of 10. Following 72 h of cultivation, a screening process was initiated employing 1 mg/ml puromycin (Sigma, #540,222). This screening procedure spanned approximately one week and served as preparation for subsequent experiments [35].

Following cell infection, a treatment involving 25 µg/ml of the PI3K activator 740 Y-P (HY-P0175, MedChemExpress) was administered for a duration of 48 h [36]. Subsequently, the subsequent testing procedures were carried out. The assessment of the knockdown effects of NRG1 was divided into three groups: NC, sh-Nrg1-1, and sh-Nrg1-2. The effects of treatment with agonists were evaluated in two groups: sh-Nrg1 and sh-Nrg1 + 740 P-Y.

### Histology and immune staining

Fresh PDX tissue samples, patient tumor specimens, and control standard tissue samples were subjected to fixation in a 10% formalin buffer solution (F5554, Thermo Fisher Scientific). Subsequently, they underwent a dehydration process, were embedded in paraffin, and were longitudinally sectioned into slices with a thickness of 5 μm. Staining of these samples was conducted using Hematoxylin (NO. E607317, Sangon Biotech) and Eosin (G1100, Solarbio) for histopathological evaluation via the Hematoxylin and Eosin (HE) staining method [34].

The specific procedure is as follows: initially, staining was carried out with 0.5% safranin for 5 min, followed by rinsing with water. Next, samples were immersed in 75% hydrochloric acid ethanol for identification, treated with ammonia for counterstaining, stained with eosin for 2 min, dehydrated using a gradient of ethanol, clarified with xylene, and finally sealed with neutral gum. Alternatively, for hydrated slices, they were subjected to heat-induced repair to reach room temperature in a 10 mM sodium citrate buffer (pH 6). Afterward, they were treated with 3% H2O2 for 10 min (for immunohistochemistry), blocked with 10% GS, and then incubated overnight at 4 °C with primary antibodies, including S100 (#PA5-78161, R, 1:400, Thermo Fisher Scientific; Abcam, ab217805), MMR/CD206 (1:200; M, R&D Systems; AF2535), CD66b (1:20000, R, Abcam, ab300122), Ki67 (#ab15580, R, 1:1000, Abcam). Subsequent to PBS washing, sections were incubated with mouse anti-HRP antibody (115-035-003, Jackson ImmunoResearch) or rabbit anti-HRP antibody (111-035-003, Jackson ImmunoResearch) for 2 h and then mounted with neutral gum for observation and photography under a microscope [29]. The signal intensity of IHC staining was quantified using the image analysis software ImageJ. The specific method involved obtaining the average intensity of the target protein DAB staining through image format conversion and comparing the relative intensity with the control signal intensity as the standard.

### Cellular immune fluorescence

The cell lines obtained from stable passages of the tumor source were seeded onto coverslips that had been pre-treated overnight at a ratio of 5000 cells per well. This pre-treatment involved the use of 1 mg/ml poly-L-lysine (Sigma, #P7890-100). The cells were fixed in 4% PFA for 20 min, followed by a 1-minute permeabilization treatment with 0.3% TritonX-100. Subsequently, they were washed in PBS and blocked for 2 h in 1% BSA. The primary antibodies, S100B (Thermo Fisher Scientific, Waltham, MA, USA, #PA5-78161, 1:400) and F-actin (Abcam, ab205, 1:200), were incubated at room temperature. After a PBS wash, incubation was performed for 1 h using Alexa Fluor-488 (Invitrogen, A-11,029, 1:500) and Alexa Fluor-546 (Invitrogen, A-21,085, 1:500) as secondary antibodies. Finally, staining was conducted using DAPI, and observation and photography were carried out under a fluorescence microscope [37].

### Western blot

Proteins were extracted from cells and tissues using RIPA lysis buffer along with PMSF (Solarbio, P0100). The protein concentration was determined using the BCA method. Subsequently, 30 µg of protein lysate was loaded onto a 10% or 12% SDS-PAGE gel. The proteins were then transferred onto a PVDF membrane (Millipore, IPFL00010) using a semi-dry transfer apparatus from Bio-Rad Laboratories. The membrane was immersed in 5% skim milk powder for at least 1 h, and the primary antibody targeting the desired protein was incubated overnight at 4 °C. Following incubation, the membrane was subjected to washes in TBST (three times for 10 min each) and probed with either mouse anti-HRP (115-035-003, Jackson ImmunoResearch) or rabbit anti-HRP (111-035-003, Jackson ImmunoResearch). Protein detection was carried out using the chemiluminescent substrate Plus (#34,580, Sigma) and imaged using an Alpha Imager HP system. Relative protein expression levels were determined by analyzing the grayscale values of the target bands and reference bands with Image software, with GAPDH serving as the internal control. The antibodies utilized included NRG1 (ab217805, Abcam), PI3K (#4257; Cell Signaling Technology), phosphor-PI3K (#4228; Cell Signaling Technology), Akt (#9272; Cell Signaling Technology), phospho-Akt (Ser473)(#4060; Cell Signaling Technology), and GAPDH (1:1000, Santa Cruz, sc-32,233) [28, 38].

### RT-qPCR

The extraction of total RNA from tissues and cells was conducted using TRIzol reagent (15,596,026, Invitrogen). Subsequently, cDNA synthesis was carried out utilizing the VBScript II kit (Abclone, RK20404), with 1 µg of total RNA as the starting material. Following this, qRT-PCR was performed on a Roche LightCycler480 high-throughput fluorescence quantitative PCR instrument (Roche, USA), in conjunction with the SYBR® Green JumpStart™ Taq ReadyMix™ kit (Roche, S5193). Each well included 3 replicates [39]. Detailed primer sequences can be found in (Table S2). The relative expression level of mRNA was assessed using the 2-ΔΔCt method, where ΔΔCt is calculated as follows: (average Ct value of the target gene in the experimental group - average Ct value of the reference gene in the experimental group) - (average Ct value of the target gene in the control group - average Ct value of the reference gene in the control group).

### Flow cytometry

The tissue was finely chopped and subjected to collagenase treatment to dissociate it into individual cells. Subsequently, the suspended cells underwent fixation with a cell-fixing solution for a duration of 15 min, followed by incubation in a solution containing a membrane permeabilization reagent and antibodies targeting CD68, CD206, CD14, and CD66b for 40 min, all carried out under dark conditions. After thorough washing and suspension, the cells were transferred to flow tubes for analysis using the FACScan flow cytometry system (Beckman, USA). Cell adhesion and debris were excluded, and the analysis focused on the CD45 positive cell gate. Within this gate, CD68 + CD206 + double-positive cells were identified as M2 macrophages. Furthermore, CD14 + CD66b + double-positive cells within the CD45 positive cell gate were recognized as neutrophils [31]. The specific antibodies used included CD45-PerCP (eBioscience, 45-0459-42), CD206-PE (eBioscience, 12-2069-42), CD68-PE-Cyanine7 (eBioscience, 25-0681-82), CD66b-APC (eBioscience, 17-0668-42), and CD14-PE (eBioscience, 12-0149-42).

Cell apoptosis was assessed utilizing the Annexin V-APC/PI apoptosis detection kit (E-CK-A217, Elabscience) [31]. Initially, the cells were suspended in PBS and subsequently incubated with Annexin V-APC and propidium iodide at room temperature in a dark environment for a duration of 15 min. Analysis of the apoptotic cells was carried out using the FACScan flow cytometry system (Becton Dickinson, USA). In the resulting analysis, the upper left corner corresponded to necrotic cells, the upper right corner denoted late-stage apoptotic cells, the lower right corner represented early-stage apoptotic cells, and the lower left corner signified cells that had not undergone apoptosis.

The cell cycle of each cell group was verified using the Cell Cycle Kit (E-CK-A351, Elabscience Biotechnology) [31]. Following the necessary processing steps, cell pellets from each group were collected and fixed overnight in 75% ethanol at 4 °C. After adjusting the cell concentration to 1 × 106 cells/ml, RNase (50 mg/ml, EN0531 from ThermoFisher) and 1 mg/ml PI (25535-16-4 from Sangon Biotech) were added, and the mixture was incubated at 37℃ for 30 min. Subsequently, the samples were transferred to flow cytometry tubes for the detection and analysis of cell proportions in the G0/G1, S, G2, and M phases of the cell cycle.

### Transwell cell migration assay

The procedure involved suspending cells in serum-free culture medium and then seeding them onto the upper chamber of a Tranwell system at a density of 1 × 105 cells per well. The Tranwell system was placed in a 24-well plate (Corning, NY, #3422), and the cells were subjected to ischemic starvation treatment for 6 h. The lower chamber contained a growth medium with 10% FBS. After incubation for 24 h, the small chamber of the Tranwell was removed, and the cells on the upper layer of the Tranwell micropores were gently wiped using a cotton swab. Subsequently, the cells were fixed in 4% formaldehyde for 1 h, followed by staining with 0.1% crystal violet (Sigma-Aldrich; Merck KGaA) at room temperature for 1 h. Excess dye was washed away with PBS, and the cells were allowed to air dry. They were then observed and photographed using a microscope. The cell quantification process involved taking photos in 5 regions, repeating each specimen 3 times for accurate quantification [35].

### Statistical analysis

Data analysis for this study was performed using SPSS software (IBM, USA, version 21.0). Measurement data were presented as mean ± standard deviation. Normality and homogeneity of variance tests were conducted as preliminary checks. For between-group comparisons, the independent t-test was employed, assuming normal distribution and equal variances. Comparison among multiple groups was conducted using either one-way ANOVA or repeated measures ANOVA, assuming normal distribution and homogeneity of variances. To explore the relationships between variables, Pearson correlation analysis was utilized. Statistical significance was set at *P* < 0.05.

## Results

### Differential expression genes were identified in BNST patient samples through transcriptome analysis

**Fig. 1:**
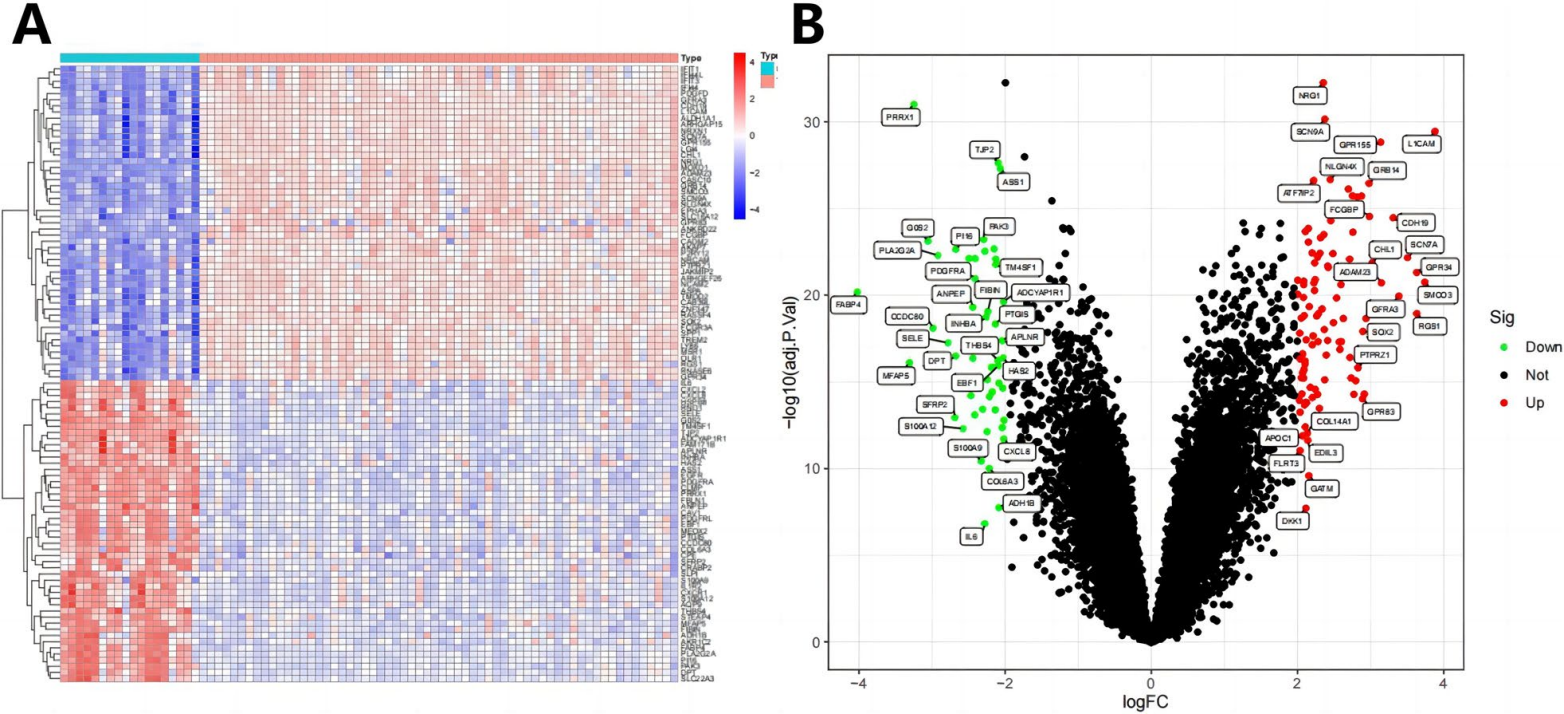
Candidate target genes were selected based on the GEO chip dataset (GSE39645 and GSE54934) related to BNST. **A **We merged the differential gene expression data from the two datasets and plotted a heatmap (GSE39645: Control group *n*  = 9, Tumor group: *n*  = 31; GSE54934: Control group *n*  = 9, Tumor group: *n*  = 31). **B **We also plotted a volcano plot of differential gene expression in the dataset. Red dots represent upregulated genes, green dots represent downregulated genes, and black dots represent genes with no difference

We studied the transcriptome RNA sequencing data of neurofibroma and control samples retrieved using the GEO database. We obtained the GSE39645 and GSE54934 datasets and merged them after batch effect removal. We used |logFC| > 2 and P value < 0.05 as thresholds to filter differentially expressed genes. The results showed that 982 upregulated genes and 375 down-regulated genes were identified (Fig. 1A, B).

### WGCNA builds gene co-expression networks to identify the critical module and its related genes

**Fig. 2:**
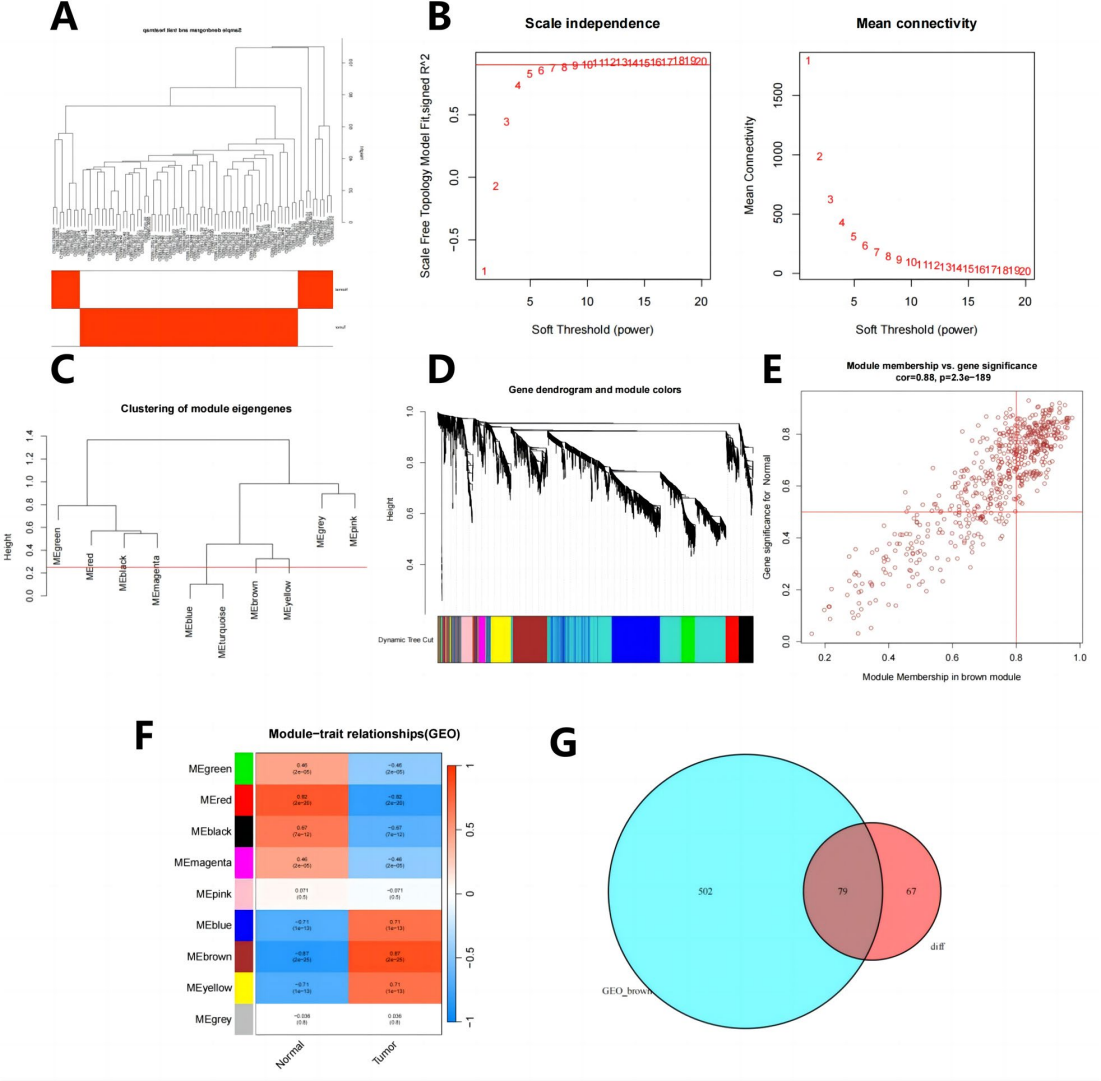
Co-expression analysis of BNST-related genes using WGCNA. **A** Cluster dendrogram of control and tumor samples of BNST patients. **B** Scale-free fit index (left) and average connectivity (right) correspond to different values of soft thresholding power β. The red line represents a correlation coefficient of 0.9. **C** Hierarchical clustering of feature gene modules summarizing the clustering analysis. **D** Dendrogram of co-expressed genes, with each leaf representing a different gene module. **E** Scatter plot showing the correlation between the brown module and tumor clinical features, with P-values shown in the Figure. **F** Heatmap of the correlations between different modules and BNST in the merged dataset. Each cell contains the corresponding correlation and *P*-value. **G** Venn diagram showing the intersection of differentially expressed genes and brown module genes

Our study employed WGCNA analysis to investigate the relationship between immune infiltration and related genes, constructing a gene co-expression network. We selected modules showing the most differences in correlation between tumor tissue and normal tissue as critical modules. By intersecting the relevant genes of critical modules and differential genes, we obtained 79 intersecting genes. After the hierarchical clustering of the samples (Fig. 2A), we select β = 10 (scale-free *R* = 0.9) as the soft threshold to construct the scale-free network (Fig. 2B). We also identified 9 co-expression modules of genes (Fig. 2C, D), among which the brown module showed the strongest correlation with tumor clinical features (Fig. 2E), with a correlation level of 0.87 (*p* < 0.05)(Fig. 2F). Therefore, we identified 581 genes in the brown module as neurilemmoma-related genes. Next, the intersection of brown module-related genes and differentially expressed genes yielded 79 intersecting genes (Fig. 2G).

### NRG1 may be a potential molecular marker for diagnostic use in BNST

**Fig. 3:**
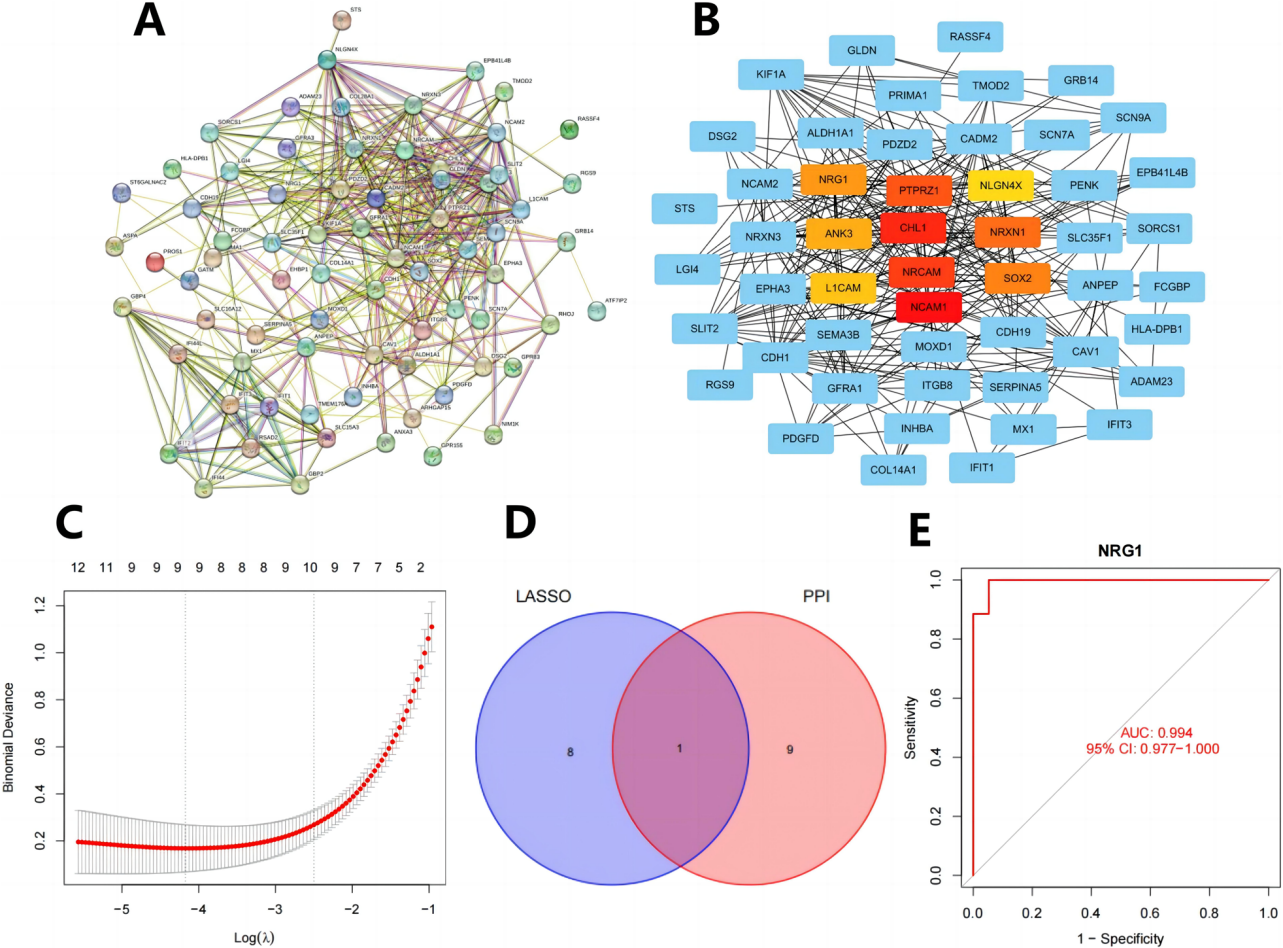
Selection of disease-specific genes for predicting BNST occurrence. **A** Construction of the intersection gene PPI network. **B** Visualization of the PPI network of hub genes and their adjacent nodes using Cytoscape software. **C **LASSO analysis results: the x-axis represents log(λ) values, and the y-axis represents Binomial Deviance. The graph shows the number of genes retained when using different log(λ) values, and the log(λ) value and number of retained genes corresponding to the optimal Binomial Deviance value are marked with dashed lines. **D **The intersection of candidate genes obtained through LASSO analysis and PPI protein interaction screening is shown by a Venn diagram. **E **Validation through the merged dataset demonstrates that NRG1 has a higher predictive value in BNST diagnosis

To further screen potential molecular biomarkers associated with the diagnosis of BNST, we imported the overlapping genes into the String database, limited to the human species, with a confidence score threshold set at 0.7. Protein-protein interactions (PPI) network for identifying differentially expressed genes (Fig. 3A). Later, we used the Cytoscape software for visualization and the cytoHubba plugin [40] to Sort and filter genes based on their MCC values to identify the top-ranking genes (NCAM1, CHL1, NRCAM, PTPRZ1, NRXN1, SOX2, NRG1, ANK3, L1CAM, NLGN4X) as hub genes. Construct a network using the hub genes and their adjacent nodes (Fig. 3B). Then, we performed LASSO regression analysis on the merged dataset and finally selected 10 candidate genes (NRG1, ATF7IP2, PDGFD, VLDLR, CASC10, PLA2G2A, FABP4, PLA2G4A, ANKRD22) (Fig. 3C). And intersect these with the candidate genes obtained from PPI network analysis to obtain a critical network containing the disease core gene NRG1 (Fig. 3D). We evaluated the predictive value of NRG1 for BNST diagnosis based on our merged dataset using ROC curve analysis.

The results indicate that NRG1, as a biomarker, has a high predictive value for BNST (AUC = 0.994, 95% CI = 0.977-1.000) (Fig. 3E). Based on the above results, NRG1 could be used as a novel biomarker for diagnosing BNST.

### Immune infiltration analysis in tumor and control samples of BNST patients with NRG1-related tumors

**Fig. 4:**
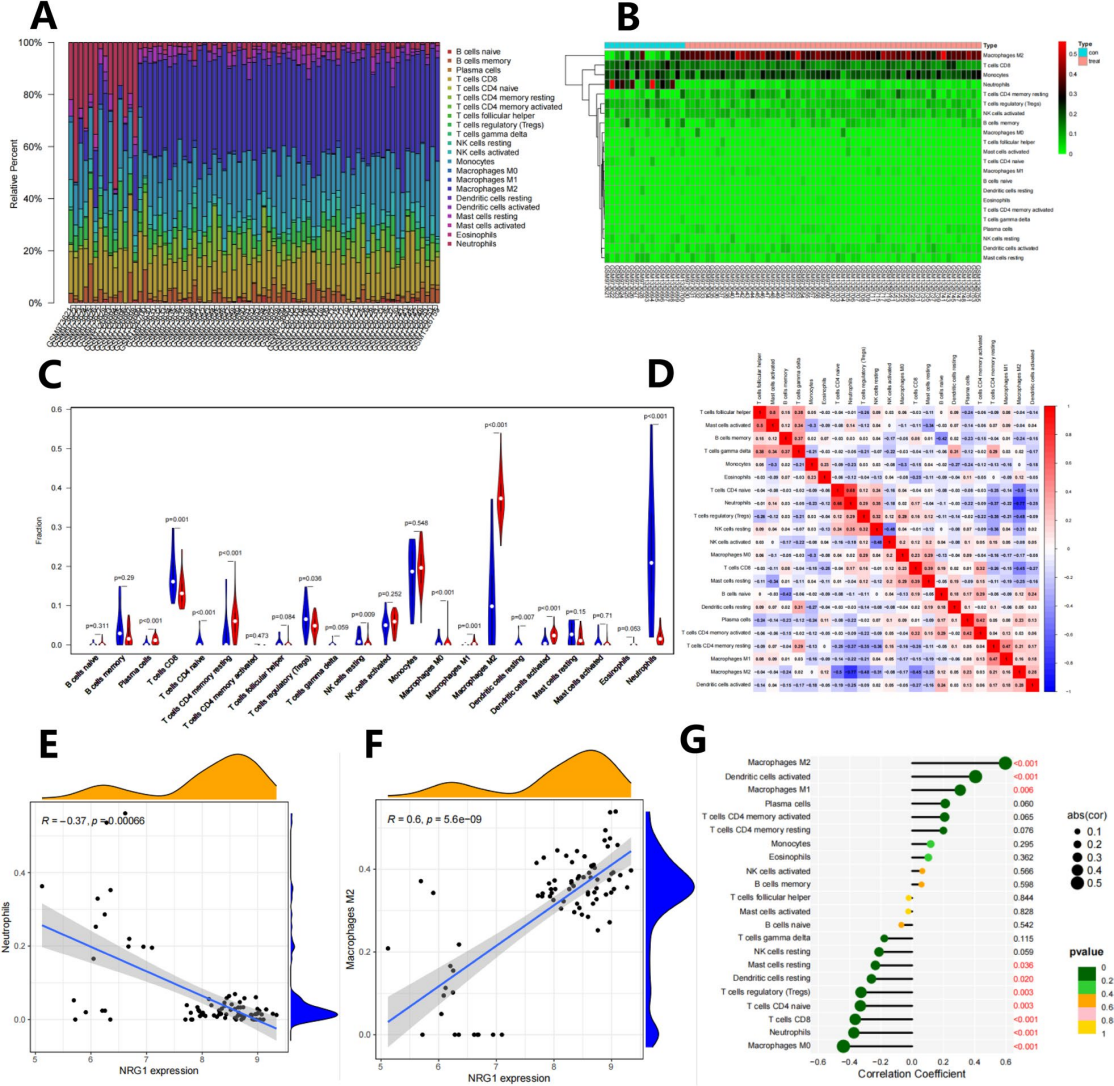
Analysis of immune cell composition and correlation in BNST samples. **A** Bar chart showing the analysis of immune cell composition in normal tissue and tumor tissue of BNST patients. The x-axis represents the sample number, and the y-axis represents the relative content of immune cells. Different colors represent different types of immune cells, with a color legend provided on the right. **B** Heatmap showing the analysis of immune cell composition in normal tissue and tumor tissue of BNST patients. The x-axis represents the sample number, and the y-axis represents different types of immune cells. Different colors in the heatmap represent the relative content of immune cells. **C** Difference plot representing the difference in immune cell composition proportion between normal tissue (blue) and tumor tissue (red) of BNST patients. **D** Immune cell correlation graph. Each small square represents the correlation between two immune cells. Positive correlations are shown in red, and negative correlations are shown in blue. The darker the color, the higher the correlation. **E**-**F** Correlation analysis of immune cell components differentially correlated with NRG1 expression. The x-axis represents the NRG1 expression level, and the y-axis represents the content of immune cells. In this case, (**E**) represents resting neutrophils, and (**F**) represents M2 macrophages. **G** Lollipop plot of the correlation analysis between all immune cell components and NRG1 expression

For the convenience of subsequent analysis, we performed immune infiltration analysis on the transcriptome RNA sequencing data of tumor and standard control samples using the CIBERSORT algorithm. After removing samples with a p-value greater than 0.05, we obtained 75 samples. The results showed no differences in the overall composition of immune cells between the tumor group and the control group of patients with neuroblastoma. However, there were differences in the proportions of different immune cell types (Fig. 4A, B): the tumor group had abnormally high levels of M2 macrophage infiltration, while the infiltration level of neutrophils was insignificant. The infiltration level of neutrophils in the control group is high, while the infiltration level of M2 macrophages is low. Further differential analysis revealed that the expression levels of M2 macrophages and neutrophils showed the most significant differences between tumor samples and standard control samples (Fig. 4C), further validating the previous findings.

Subsequently, a correlation analysis of the immune cell composition was conducted on 75 selected samples, revealing a certain degree of correlation between immune cells (Fig. 4D). This correlation was mostly observed between different types of immune cells, particularly showing a strong negative correlation between M2 macrophages and neutrophils (*r*=-0.77, *p* < 0.05). To further understand the correlation between NRG1 and immune cell composition in BNST patient samples, we performed a correlation analysis of all immune cell components and NRG1 expression levels. correlation was found between the expression levels of M2 macrophages, neutrophils, and NRG1, as shown in Fig. 4E, F (*p* < 0.05). Specifically, there is a positive correlation between M2 macrophages and NRG1 expression, while there is a negative correlation between neutrophils and NRG1 expression. Besides, there is also a correlation between the expression levels of dendritic cells activated, macrophages M1, T cells CD and NRG1 (Fig. 4G, *p* < 0.05).

These results indicate that NRG1 may be involved in the infiltration of immune cells in the blood immune environment of BNST patients, thereby affecting the progression of BNST patients’ condition.

### NRG1 may be involved in forming BNST by influencing the PI3K-Akt pathway

**Fig. 5:**
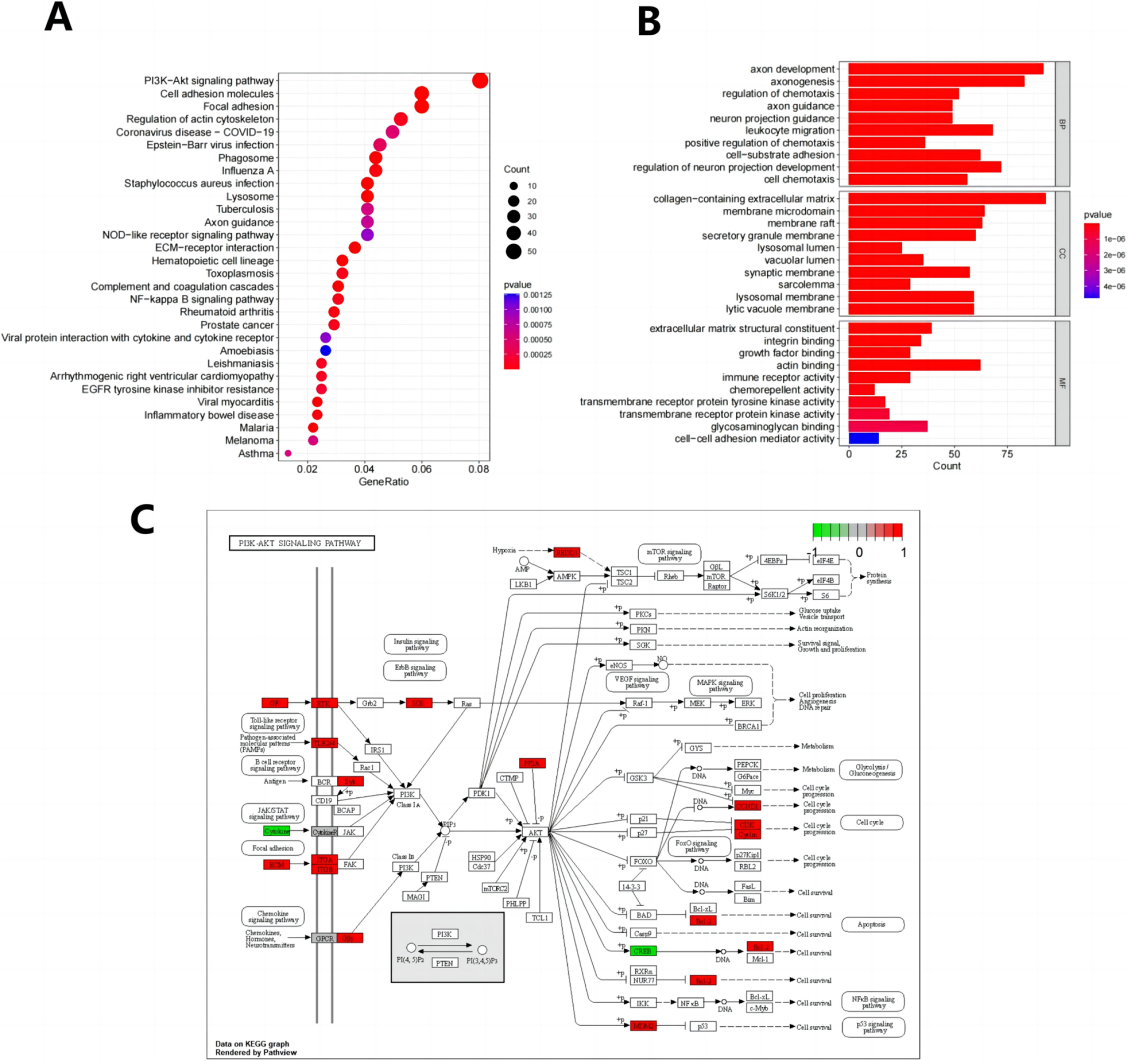
Functional exploration of NRG1 in BNSTs. **A **Presentation of GO functional analysis results of Differentially Expressed Genes (DEGs) at the biological process (BP), cellular component (CC), and molecular function (MF) levels. **B **Results of KEGG pathway enrichment analysis of DEGs

To delve into the function of NRG1 in the BNST, we integrated the differentially expressed genes obtained from the dataset with the essential module genes derived from the gene co-expression network established via WGCNA. Subsequently, we conducted GO functional analysis and KEGG pathway analysis on this combined set of genes. The GO functional analysis results revealed that the shared genes were prominently enriched in various biological processes (BP) including axon development, axonogenesis, and regulation of neuron projection development. In terms of cellular components (CC), these genes exhibited enrichment in categories such as collagen-containing extracellular matrix, membrane microdomains, membrane rafts, and secretory granule membranes. Moreover, the molecular functions (MF) of these genes were notably associated with functions such as extracellular matrix structural constituents, integrin binding, growth factor binding, and actin-binding (as illustrated in Fig. 5A). In parallel, the KEGG pathway analysis indicated that the common genes were significantly enriched in pathways such as the PI3K-Akt signaling pathway, cell adhesion molecules, and focal adhesions (Fig. 5B).

The functional enrichment results suggest that the intersected genes are mainly involved in axon development, axon generation, and regulation of neuronal projection development. They also function in collagen-containing extracellular matrix, membrane microdomains, membrane rafts, and secretory granule membranes. In addition, intersectin genes are involved in processes such as extracellular matrix composition, integrin binding, growth factor binding, and actin binding. According to the analysis results of KEGG, the intersection genes were involved in essential life processes such as the PI3K-Akt signaling pathway, cell adhesion molecules, and focal adhesions.

Abnormal activation of the PI3K-Akt signaling pathway has been reported in the literature to be the molecular basis of neurofibromatosis tumorigenesis, and inhibiting this pathway is a potential therapeutic strategy [29, 38]. In addition, previous studies have found that in preclinical cell line models of neurofibromas, inhibiting the PI3K-Akt signaling pathway through drug therapy could attenuate tumor growth [28]. We identified an overlap between the intersection genes and 18 genes in the PI3K-Akt signaling pathway (Fig. 5C).

Based on the above results, NRG1 may play a key role in tumor cell growth, proliferation, and survival by influencing the formation of BNST through the PI3K-Akt signaling pathway.

### Quality control, filtering, and principal component analysis of scRNA-seq data from cancer tissues of BNST patients

**Fig. 6:**
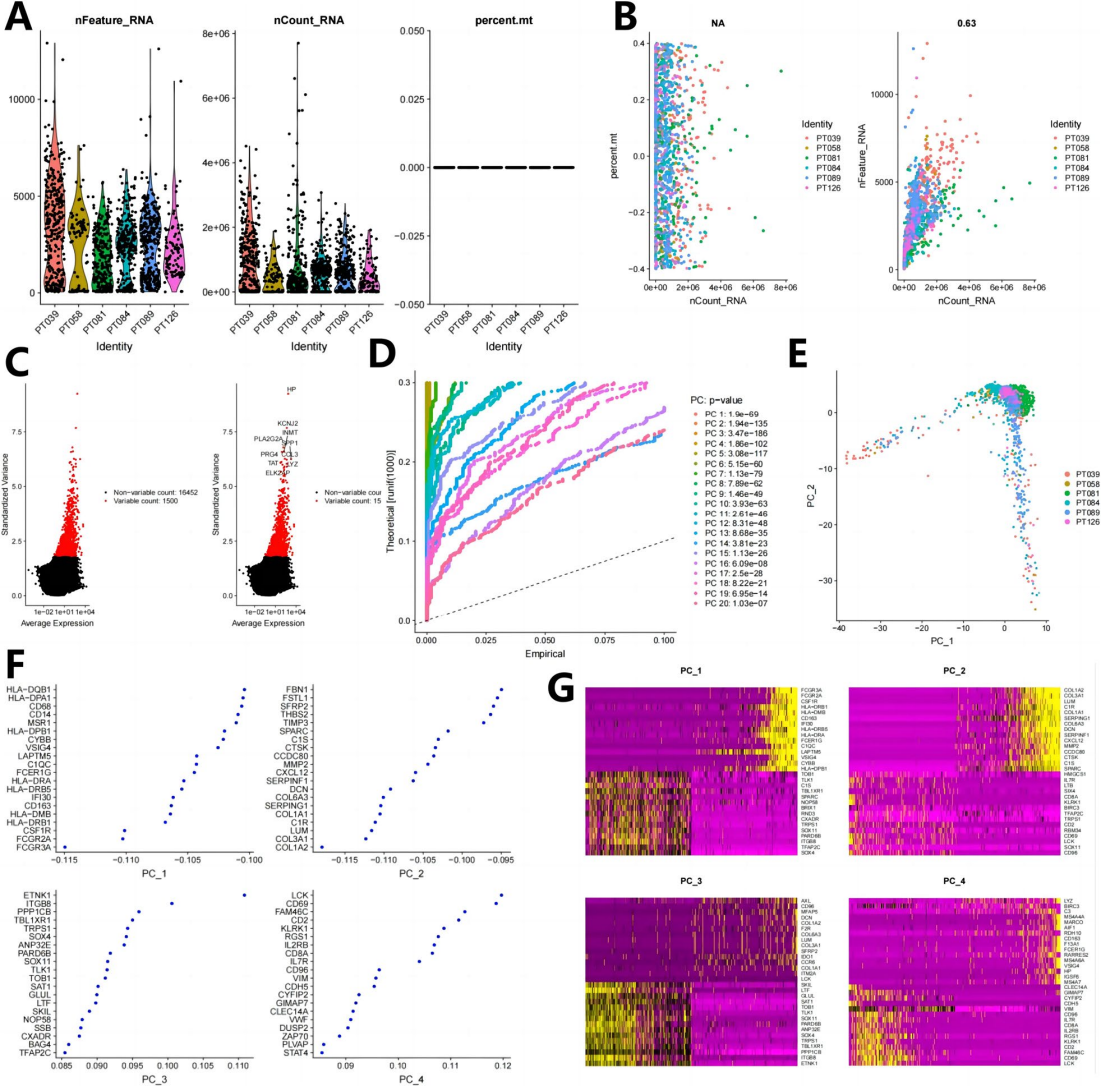
Quality control, filtering, and principal component analysis of scRNA-seq data. **A **Quality control was performed on 2012 cells from three BNST patient tissue samples. The three scatter plots show the number of nFeature_RNA, nCount_RNA, and percent. Mt in each cell. **B **Correlation between nCount and percent.mt (left plot), and correlation between nCount and nFeature (right plot). **C **Highly variable genes in the samples were selected through variance analysis (red dots represent highly variable genes, black dots represent unchanged genes). **D ***P*-values of PCA analysis results for 15 PCs. **E **Performance of cells from different sample sources in PCA principal component analysis (each dot represents a cell, and different colors represent different samples). **F**-**G **Heatmaps of feature genes and their expression levels based on the first two PCs of PCA analysis

To further screen for cell types involved in BNST formation and development and closely associated with candidate genes, we collected BNST samples for single-cell RNA sequencing. After quality control and normalization of scRNA-seq data using the “Seurat” package in R software, we obtained a graph illustrating the distribution of cell RNA (Fig. 6A). The correlation coefficients indicate a correlation of *r* = 0.05 between nCount and percent. Mt, and a correlation of *r* = 0.94 between nCount and nFeature, indicating a high quality of filtered cells (Fig. 6B). In identifying highly variable genes from the filtered 2012 cells, we selected the top 1500 genes with the highest variance for gene expression variance analysis and ultimately included 11,875 genes (Fig. 6C). The above results indicate that there are large numbers of highly variable genes in the BNST organization.

Subsequently, we proceeded to reduce the dimensionality of the PCA analysis results by selecting the top 1500 genes based on descending order sorting. This selection aimed to streamline the subsequent TSNE clustering of cells. The PCA analysis yielded a total of 15 principal components (PCs), which were subsequently utilized for TSNE clustering and cell annotation. To identify the “important” principal components (PCs), we assessed their significance by examining the p-values associated with each PC. Visualizing the various principal components using the JackStrawPlot function allowed us to compare the positions of the p-value distributions for each PC in relation to the mean distribution. In general, essential PCs exhibited smaller p-values, as indicated by the solid line above the dashed line, signifying their effectiveness in capturing information from the highly variable genes selected earlier (see Fig. 6D). Furthermore, we also presented the results of PCA principal component analysis for each cell sample (see Fig. 6E), as well as the feature genes associated with the first two PCs (see Fig. 6F) and their corresponding expression heatmap (see Fig. 6G).

The above results indicate that the PCA analysis is relatively reliable and could be used for subsequent cell clustering.

### Annotated 7-cell clusters from the BNST single-cell sequencing dataset

**Fig. 7:**
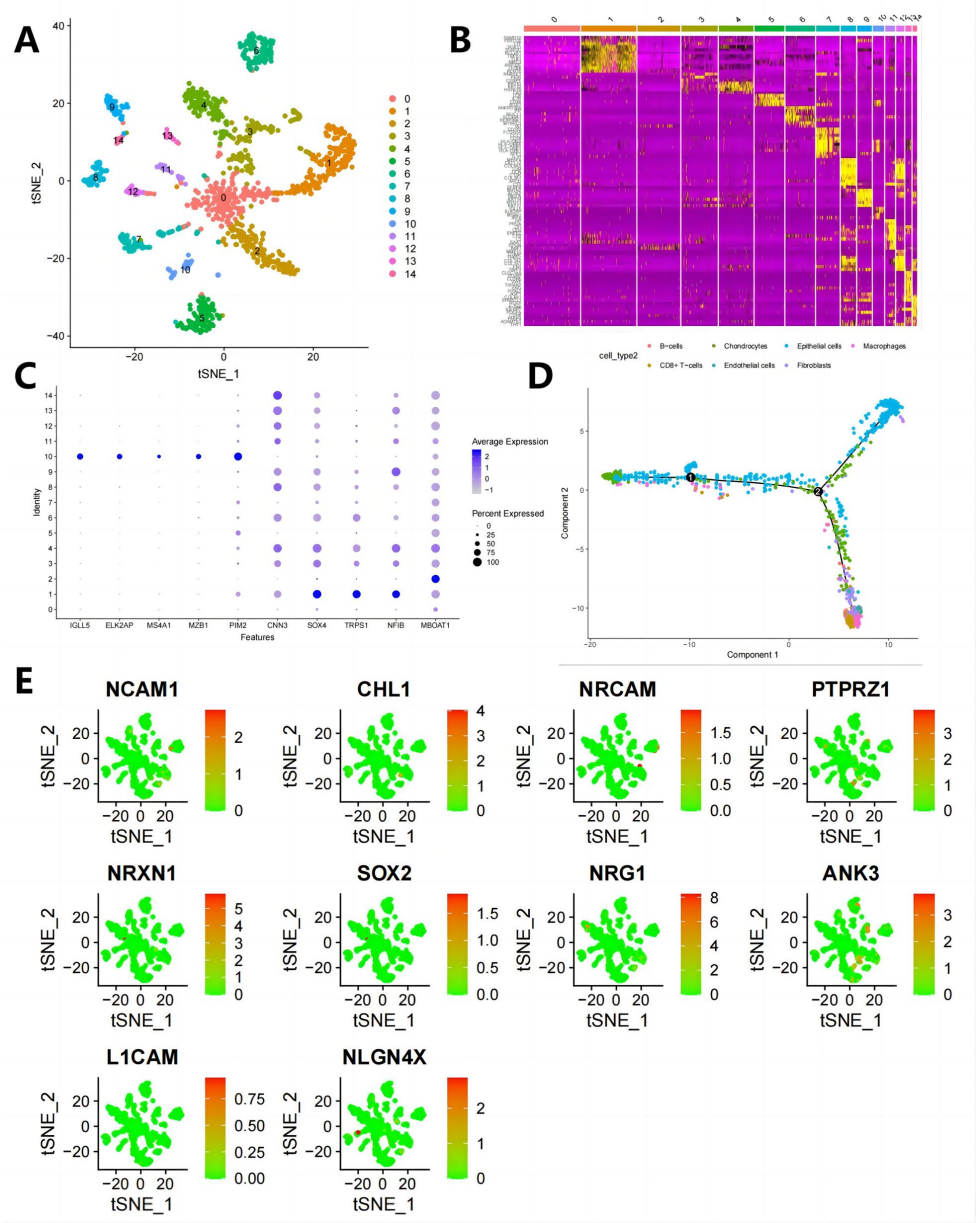
Cell clustering and expression of candidate genes in different immune cells based on scRNA-seq data. **A **Cell clustering analysis using t-SNE resulted in 16 cell clusters. **B **Heatmap of top 10 marker gene expression in cell clusters. **C **Expression of known cell lineage-specific marker genes in different clusters. The darker blue represents higher average expression levels, and the larger circle represents more cells expressing the gene. **D **Cell trajectory based on cell types. **E **Distribution and expression of top 10 candidate genes in immune cells based on PPI interaction scoring

After TSNE clustering analysis, we divided all cells into 14 clusters (Fig. 7A). We obtained the characteristic genes of each cluster of cells and generated expression heat maps for the top 10 marker genes in these 14 cell clusters. The marker genes for macrophages include APOC1, C1QB, APOE, C1QA, C1QC, TYROBP, CCL3, LYZ, AIF1, and HLA-DRA. The marker genes for endothelial cells are CLDN5, ITM2A, and VWF. The marker genes for epithelial cells are FGG and SCGB3A1. The marker genes for NK cells are PRF1, CD3E, and CD7. The marker genes for tissue stem cells are TAGLN and DCN (Fig. 7B, C).

To select crucial cells, we utilized the “monocle” package for pseudo-temporal analysis. The temporal analysis aims to construct cellular lineage development based on the temporal variation of gene expression levels in different subpopulations of cells. Virtual time reconstructs cells’ developmental, aging, and disease progression trajectories through dynamic changes in gene expression information, revealing the evolutionary state and patterns of cells and analyzing the associated driving gene expression characteristics. Based on the expression trends of sorted genes, we sorted cells and completed trajectory construction (Fig. 7D).

We further analyzed the distribution and expression of the top 10 candidate genes involved in PPI interactions in immune cells (Fig. 7E). NCAM1 is less distributed in the BNST, mainly in T cells, epithelial cells, and fibroblasts, with low expression levels. CHL1 is mainly distributed in T cells, expressed highly in BNST, and found to be highly expressed in adverse prognoses of BNST patients. Therefore, we speculate that NRCAM may promote BNST progression by influencing T cell-mediated tumor immune responses to cancer cells.

PTPRZ1 and NRXN1 were observed to be present in 14 distinct cell clusters, while SOX2 exhibited widespread distribution across these 14 cell clusters, albeit with notably elevated expression levels detected in thyroid follicle cells. NRG1, on the other hand, displayed a predominant presence in myeloid cells and T cells. ANK3 exhibited a distribution pattern encompassing T cells, endothelial cells, thyroid follicle cells, and fibroblasts, with thyroid follicle cells showing a higher expression level. L1CAM and NLGN4X were identified in all 7 cell clusters, with a relatively higher occurrence in myeloid cells. These findings collectively suggest that these 10 candidate genes are predominantly localized within T cells and myeloid cells, thereby hinting at a potential association between the progression of BNST and the enrichment of these genes in T cells and myeloid cells.

### BNST clinical tumor samples display high expression of NRG1 and immune microenvironment remodeling effect

**Fig. 8:**
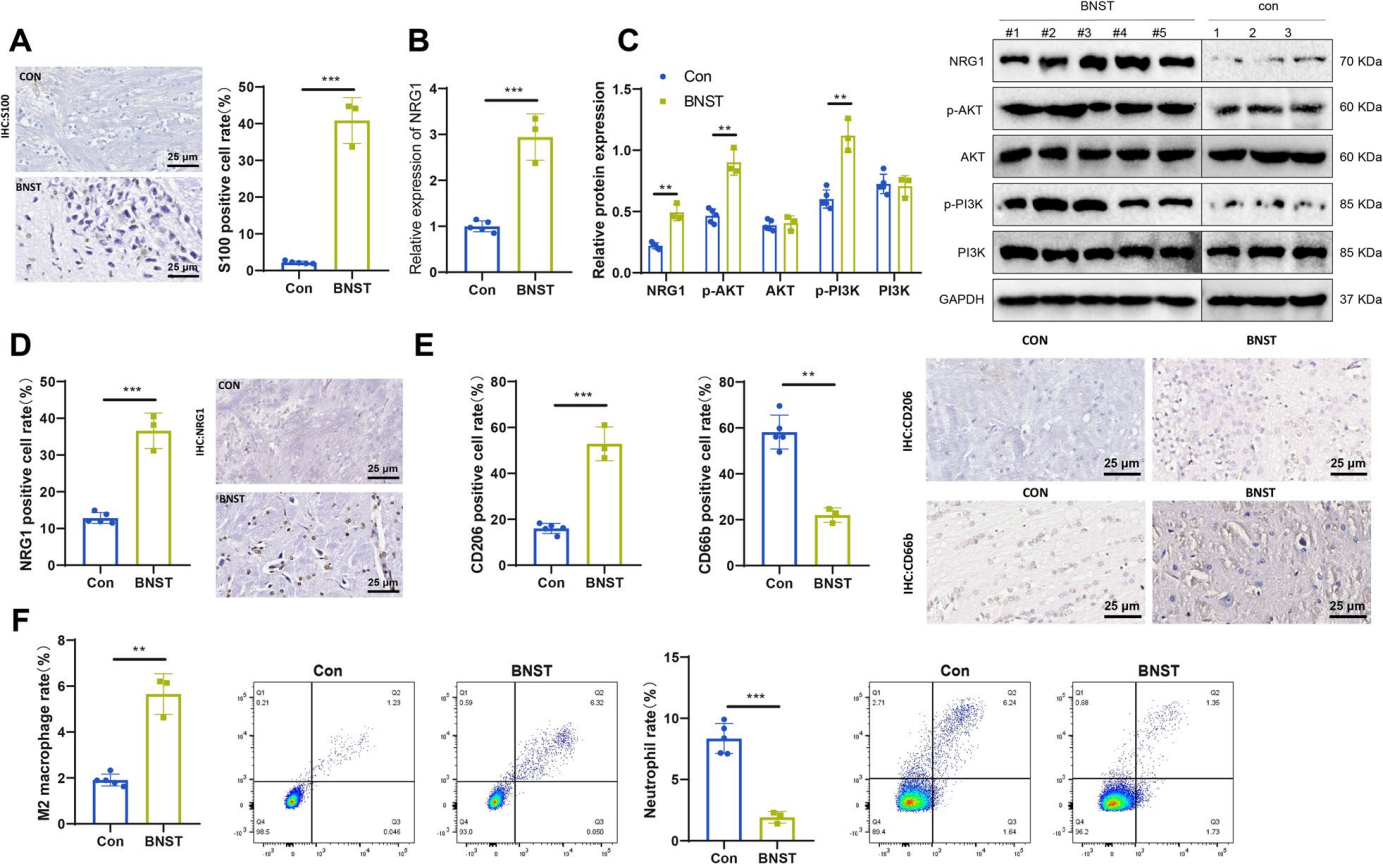
Detection of NRG1, immune cells influenced by NRG1, and PI3K/AKT in clinical tissue samples. **A **IHC detection of S100 expression in clinical tissue samples. con: normal vestibular tissue. S100 is mainly expressed in the BNST cytoplasm. S100 is a biological marker of BNST—scale bar: 20 μm. The statistical graph is on the right. **B **RT-qPCR detection of Nrg1 mRNA expression levels in clinical tissue samples. **C **Western blot detection of NRG1 and key proteins in the PI3K/AKT pathway in clinical tissue samples. The statistical graph is on the right. **D **IHC detection of NRG1 expression in clinical tissue samples. Mainly distributed in the cytoplasm and membrane of myelin-forming cells and macrophages. Scale bar: 20 μm. The statistical graph is on the right. **E **Changes in M2 macrophages (M2 mac) and neutrophils (neu) in clinical samples detected by IHC. CD206-scale bar: 20 μm. CD66b: scale bar: 20 μm. Both are expressed on the cell membrane of macrophages and neutrophils. Statistical graphs on the right. **F **Changes in M2 macrophages and neutrophils in clinical samples detected by flow cytometry. M2 mac: CD68 + CD206 + , neu: CD14 + CD66b + . Statistical graph in the first quadrant. con group: *n*  = 3; BNST group: *n*  = 5. Indicates a difference compared to con, where *P*  < 0.05 indicates a difference compared to con, *P*  < 0.01, and *** indicates a difference compared to con, *P*  < 0.0001

The conclusions obtained through database analysis need further organization. In BNST patients, high expression of NRG1 activates the PI3K/AKT signaling pathway, recruiting M2 macrophages to the tumor site and accelerating tumor progression. We collected 5 clinical samples of BNST and 3 samples of normal vestibular nerve tissue to validate these conclusions. As shown in Fig. 8A, the BNST marker S100 exhibited positive staining in immunohistochemistry. Compared to the standard control group, the expression of NRG1 in the BNST samples increased at the mRNA level (Fig. 8B) and the protein level (Fig. 8C, D).

At the same time, the phosphorylation levels of critical proteins in the PI3K/AKT signaling pathway, namely PI3K and AKT, also increased (Fig. 8C). These results suggest that high expression of NRG1 may act by activating this signaling pathway. Through immunohistochemical staining with CD206 and CD66b (Fig. 8E), as well as flow cytometric analysis of CD68 + CD206+ (M2 macrophages) and CD14 + CD66b+ (neutrophils) (Fig. 8F), we found that BNST tumor samples exhibited a higher accumulation of M2 macrophages (M2 mac) and a lower presence of neutrophils (neu). The results are consistent with the previous bioinformatics analysis, indicating a positive correlation between the high expression of NRG1 in BNST and the aggregation of M2 macrophages in the tumor site while being negatively correlated with neutrophils.

### Using the PDX model and in vitro cell lines, we validate the relationship between NRG1 and PI3K/AKT in the BNST

To further elucidate the role of NRG1 in BNST, we followed the protocol outlined in Figure S1A to dissociate human BNST tumor tissue into single cells and then transplanted them onto the mouse sciatic nerve after in vitro culture, thus establishing a PDX model [29].

First, by comparing the conditions of the original tumor tissue and the xenograft, it was found that both had noticeable spindle cell changes, Verocay bodies, and perivascular hyalinization (Figure S1B). At the same time, they showed positive staining in S100, and it is worth mentioning that the expression of NRG1 has been maintained at a high level (Figure S1C). In addition, Ki67 expression in heterogenous transplants is increased to some extent, which is necessary for tumor formation in vivo (Figure S1C). At the same time, the proportion of M2 macrophages and neu cells in both the primary tumor tissue and xenografts is similar (Figure S1D, E).

Furthermore, compared to normal vestibular tissue (con), critical proteins in the PI3K/AKT signaling pathway exhibit a stable high expression level (Figure S1F). These results demonstrate that xenografts maintain the same morphological and histological characteristics as the original tumor tissue, serving as a source for subsequent in vitro cell line construction. The cell line constructed in vitro exhibited high levels of S100 expression, similar to the original tumor tissue (Figure S1G).

Therefore, constructing the PDX model and in vitro cell lines provides a reliable material source for subsequent experiments.

### NRG1 maintains the characteristics and malignant behavior of BNST tumor cells through the PI3K/AKT signaling pathway

**Fig. 9:**
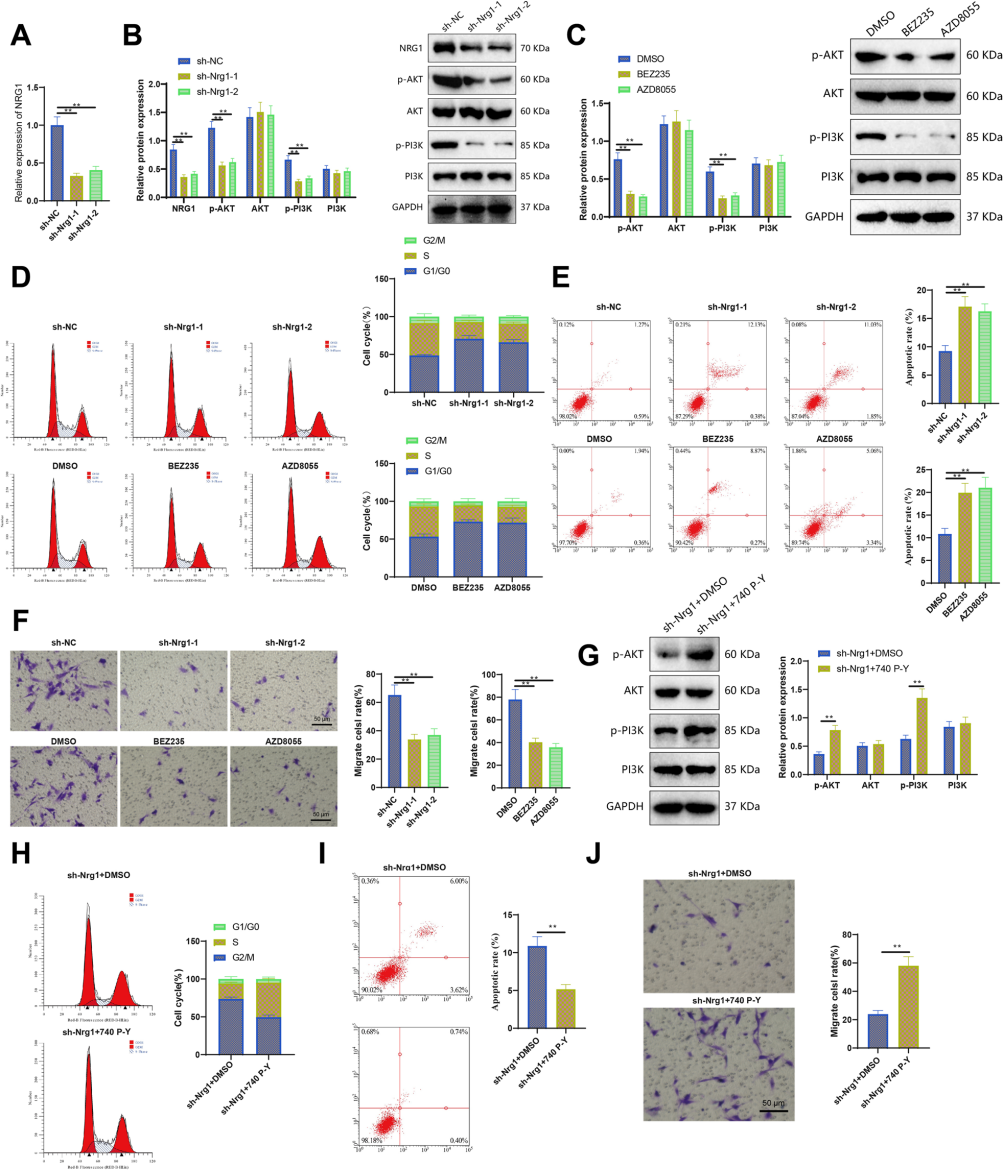
Validation of NRG1-mediated cell behavior through the PI3K/AKT signaling pathway using cell line models. **A** RT-qPCR detection of NRG1 knockdown efficiency. **B** Western blot detection of NRG1 knockdown efficiency and expression levels of critical proteins in the PI3K/AKT signaling pathway. Statistical graphs on the right. **C** Western blot detection of critical proteins in the PI3K/AKT signaling pathway in cells treated with a PI3K inhibitor. Statistical graphs on the right. **D** Cell cycle of cells after NRG1 knockdown and PI3K inhibitor treatment detected by flow cytometry. Statistical graphs on the right. **E** After NRG1 knockdown and PI3K inhibitor treatment, apoptosis proportions of cells were detected by flow cytometry. Statistical graph in the first quadrant. **F** Cell migration ability after NRG1 knockdown and PI3K inhibitor treatment detected by Transwell assay. **G** Expression levels of critical proteins in the PI3K/AKT signaling pathway in cells treated with a PI3K agonist after NRG1 knockdown detected by Western blot. Statistical graphs on the right. **H** Cell cycle changes of cells after NRG1 knockdown and PI3K agonist treatment detected by flow cytometry. Statistical graphs on the right. **I** After NRG1 knockdown and PI3K agonist treatment, apoptosis of cells was detected by flow cytometry. Statistical graph in the first quadrant. **J** Cell migration ability after NRG1 knockdown and PI3K agonist treatment detected by Transwell assay. Indicates a difference compared to sh-NC or con or sh-Nrg1 + DMSO, where *P*  < 0.05, indicates a difference compared to sh-NC or DMSO or sh-Nrg1 + DMSO, *P*  < 0.01, and indicates a difference compared to sh-NC or DMSO or sh-Nrg1 + DMSO, *P*  < 0.001, ** indicates a difference compared to sh-NC or DMSO or sh-Nrg1 + DMSO, *P*  < 0.0001

After confirming the reliability of the PDX model and cell lines, to further demonstrate that NRG1 could recruit M2 macrophages and play a role in BNST by activating the PI3K/AKT signaling pathway, we first conducted in vitro validation using cell lines. First, we established stable BNST cells with reduced NRG1 expression through virus infection. Following the confirmation of decreased efficiency of NRG1 knockdown (Fig. 9A, B), it was observed through RT-qPCR and Western blot analysis that there was a concurrent downregulation of the critical proteins p-AKT and p-PI3K (Fig. 9B). This result is equivalent to the effect of using PI3K signaling inhibitors (BEZ235 and AZD8055). Adding BEZ235 and AZD8055 could inhibit the phosphorylation of essential proteins in the PI3K/AKT signaling pathway, as shown in Fig. 9C, compared to adding the solvent group DMSO. It antagonizes the activation of this signaling pathway. By employing flow cytometry to assess the cell cycle and apoptosis of BNST cells and employing the Transwell assay to measure cell migration, experimental results showed that both knocking down NRG1 and utilizing a PI3K inhibitor resulted in a decreased proportion of cells in the S phase, indicating a deceleration in proliferation.

Furthermore, increased cell apoptosis and reduced cell migration were observed (Fig. 9D). Therefore, the knockdown of NRG1 and the inhibition of PI3K signaling activity have similar effects on BNST cells, suggesting that the PI3K/AKT signaling pathway plays a crucial role in mediating the effects of NRG1 on BNST. To verify this hypothesis, we treated NRG1 cell lines with the PI3K activator 740 P-Y for 48 h and then detected the cell cycle and apoptosis of BNST cells using flow cytometry, as well as cell migration through Transwell experiments. The experimental results showed that compared with the solvent group (sh-Nrg1 + DMSO), the sh-Nrg1 + 740 P-Y group had faster cell proliferation, reduced apoptosis, and increased migration (Fig. 9G). PI3K agonists, to some extent, rescue the phenotype of decreased cell proliferation, increased apoptosis, and decreased migration caused by the knockdown of NRG1. In conclusion, these results demonstrate that NRG1 maintains the characteristics and malignant behavior of BNST tumor cells through the PI3K/AKT signaling pathway.

### The high expression of NRG1 in BNST promotes the progress of BNST

The PDX model constructed using NOD/SCID mice is ideal for studying tumor immunity [41]. Meanwhile, our preliminary research has confirmed the reliability of establishing PDX models using primary cells derived from BNST tumors. In other words, heterografts could preserve the histopathological features of the original tumor tissue (Figure S1).

**Fig. 10:**
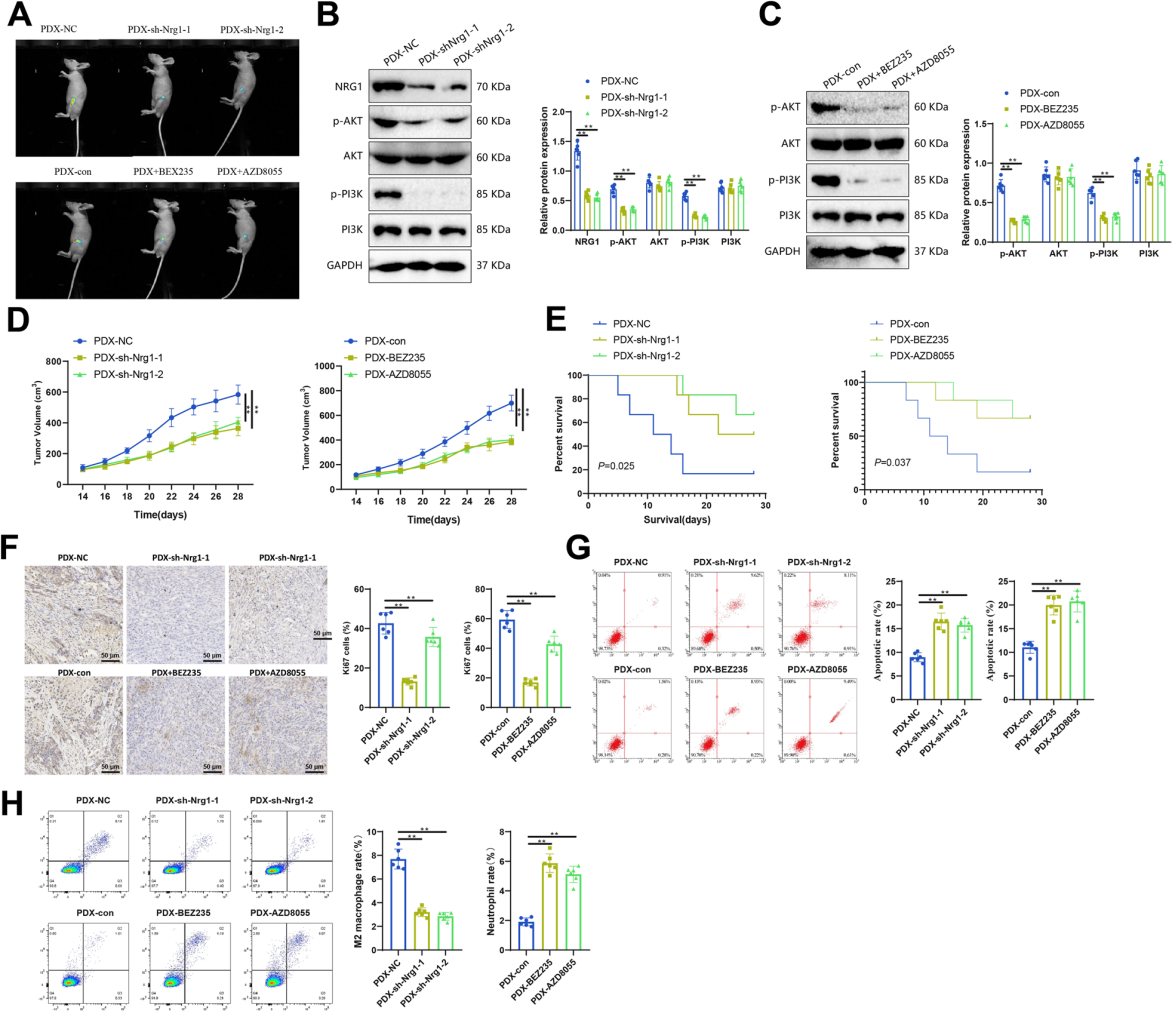
Validation of NRG1-mediated BNST progression through the PI3K/AKT signaling pathway using PDX models. **A **In vivo fluorescence imaging representation of tumor growth after sciatic nerve heterotopic transplantation. **B **Changes in critical proteins in the PI3K/AKT signaling pathway detected by Western blot in xenografts. **C **Statistical graph of protein expression in Figure B. **C** Tumor growth curve of PDX mice. **D **Kaplan-Meier survival curve of PDX mice. **F **Representative images of Ki67 IHC staining in xenografts. Scale bar: 100 μm. Ki67 is mainly expressed in cell nuclei. **G **Apoptosis of cells in xenografts detected by flow cytometry. Statistical graph in the first quadrant. **H **Flow cytometry detected the proportion of M2 macrophages (M2 mac) and neutrophils (neu) in xenografts—statistical graph in the first quadrant. M2 mac: CD68 + CD206 + , neu: CD14 + CD66b + ; *n*  = 3 per group. Indicates a difference compared to PDX-NC or PDX-con, where *P*  < 0.05 indicates a difference compared to PDX-NC or PDX-con, *P*  < 0.01 indicates a difference compared to PDX-NC or PDX-con, *P* < 0.001, ** indicates a difference compared to PDX-NC or PDX-con, *P*  < 0.0001

To demonstrate the close relationship between the influence of NRG1 on the BNST microenvironment and the activation of the PI3K/AKT pathway, we treated mice with a PI3K signaling inhibitor during tumor formation. Firstly, the formation of the tumor was demonstrated using in vivo fluorescence imaging (Fig. 10A). After using Western blot to confirm the downregulation of critical proteins in the PI3K/AKT signaling pathway (Fig. 10B, C), evaluation of the tumor revealed that the use of inhibitors reduced tumor volume (Fig. 10A and D) and improved the survival rate of mice (Fig. 10E).

Using IHC and flow cytometric analysis, the proliferation and apoptosis of xenografts were detected separately. The results showed that compared to the control group, the use of inhibitors slowed the proliferation rate of tumor cells and increased the apoptosis ratio (Fig. 10F, G). It may be the reason for the decrease in tumor volume after inhibiting the PI3K signal. Meanwhile, we used flow cytometry to detect the changes in these two cell populations to confirm the effects of NRG1 on M2 macrophages and neu cells in the immune microenvironment. The use of inhibitors decreased the proportion of M2 macrophages and increased the proportion of neu cells (Fig. 10H). This is consistent with the previous bioinformatics analysis results, indicating that the expression of NRG1 is positively correlated with M2 macrophages and negatively correlated with neu cells.

In vitro, experiments have demonstrated that downregulation of NRG1 could inhibit the malignant behavior of tumor cells. We used sh-NC cells, sh-Nrg1-1 cells, and sh-Nrg1-1 cells to construct a PDX model. The results showed that knocking down NRG1 not only resulted in a decrease in the expression levels of critical proteins in the PI3K signaling pathway (Fig. 10B, C) but also weakened tumor growth ability (Fig. 10D), leading to an extension of the survival time of mice (Fig. 10E). Consistent with the results of in vitro experiments, in NRG1 knockdown tumors, cell proliferation slows down and apoptosis increases (Fig. 10F, G).

The above results indicate that high expression of NRG1 in BNST could change the immune microenvironment, recruit M2 macrophages, regulate the ratio of neu cells by activating the PI3K/AKT signaling pathway, and promote the progression of BNST. This finding suggests that NRG1 may serve as a novel diagnostic biomarker for BNST and provides new insights into the diagnosis and treatment of this disease.

## Discussion

Diagnosing BNST has always been challenging, particularly due to the lack of specific biomarkers [42]. Traditional diagnostic methods frequently have limitations and are unable to accurately distinguish BNST from other related disorders [43]. In order to enhance the diagnosis of this disease, researchers have initiated the quest for pertinent biomarkers [44]. This study has identified NRG1 as a potential hub gene in the BNST by utilizing the GEO database alongside WGCNA and LASSO regression analysis among a multitude of genes. NRG1 offers distinct advantages compared to other recognized markers, as it demonstrates a more specific expression pattern in patient samples from the BNST [15]. Furthermore, the function and regulatory network of NRG1 are intricately associated with the pathogenesis of BNST. Hence, the identification of NRG1 offers a novel and dependable biomarker for diagnosing BNST, potentially reshaping current diagnostic approaches.

The PI3K/AKT signaling pathway serves as a crucial regulatory mechanism for essential physiological processes, including cell survival, proliferation, and metabolism. Moreover, it plays a critical role in the initiation and progression of numerous tumors [45–47]. Aberrant activation of this pathway can promote tumor proliferation, resistance to apoptosis, and enhance migration capability [48]. The research on the BNST has revealed a potential association between NRG1 and the PI3K/AKT signaling pathway [49]. This type of interaction not only regulates the proliferation and survival of cells, but also influences the immune microenvironment, particularly the infiltration and function of M2 macrophages and neutrophils [50]. By studying in vitro cell line models, it has been discovered that the downregulation of NRG1 or the use of PI3K/AKT inhibitors can effectively impede cell proliferation and migration, while inducing apoptosis [51]. In contrast, the utilization of PI3K/AKT signaling agonists enhances the proliferative and migratory abilities of cells, while decreasing the rate of apoptosis [52]. It should be noted that the PDX model has undergone consistent validation against experimental results obtained from in vitro cell lines, enhancing the reliability of these findings in multiple aspects. Therefore, NRG1 likely plays a pivotal role in BNST development via the PI3K/AKT signaling pathway, offering novel insights for future therapeutic approaches.

The immune microenvironment plays a critical role in tumor formation and development by involving various types of immune cells that interact with tumor cells, collectively influencing tumor progression [53]. The results from PDX models and tumor-derived cell lines showed consistency, indicating that knocking down NRG1 or using PI3K/AKT inhibitors inhibited cell proliferation and migration and promoted apoptosis, while the use of PI3K/AKT activators accelerated cell proliferation, increased migration ability, and decreased apoptosis rate. The experimental results of PDX models and in vitro cell lines were consistent, demonstrating that knocking down NRG1 or PI3K/AKT inhibition reduced tumor size, extended survival, slowed cell proliferation, increased apoptosis, reduced M2 macrophages, and increased neutrophils. This study unequivocally demonstrates the significant role of NRG1 in this process. Knocking down NRG1 or using PI3K/AKT inhibitors resulted in a decrease in tumor count and an increase in overall survival in PDX mouse models [51]. Specifically, the proliferative capacity of cells decreases, the rate of apoptosis increases, while the number of M2 macrophages decreases, and the number of neutrophils increases [54]. These findings elucidate the pivotal role of NRG1 in regulating the immune microenvironment in BNST patients, specifically in regard to its influence on immune cell infiltration [15]. This study provides a comprehensive understanding of the pathogenesis of BNST and suggests innovative avenues for future therapeutic interventions [55].

**Fig. 11:**
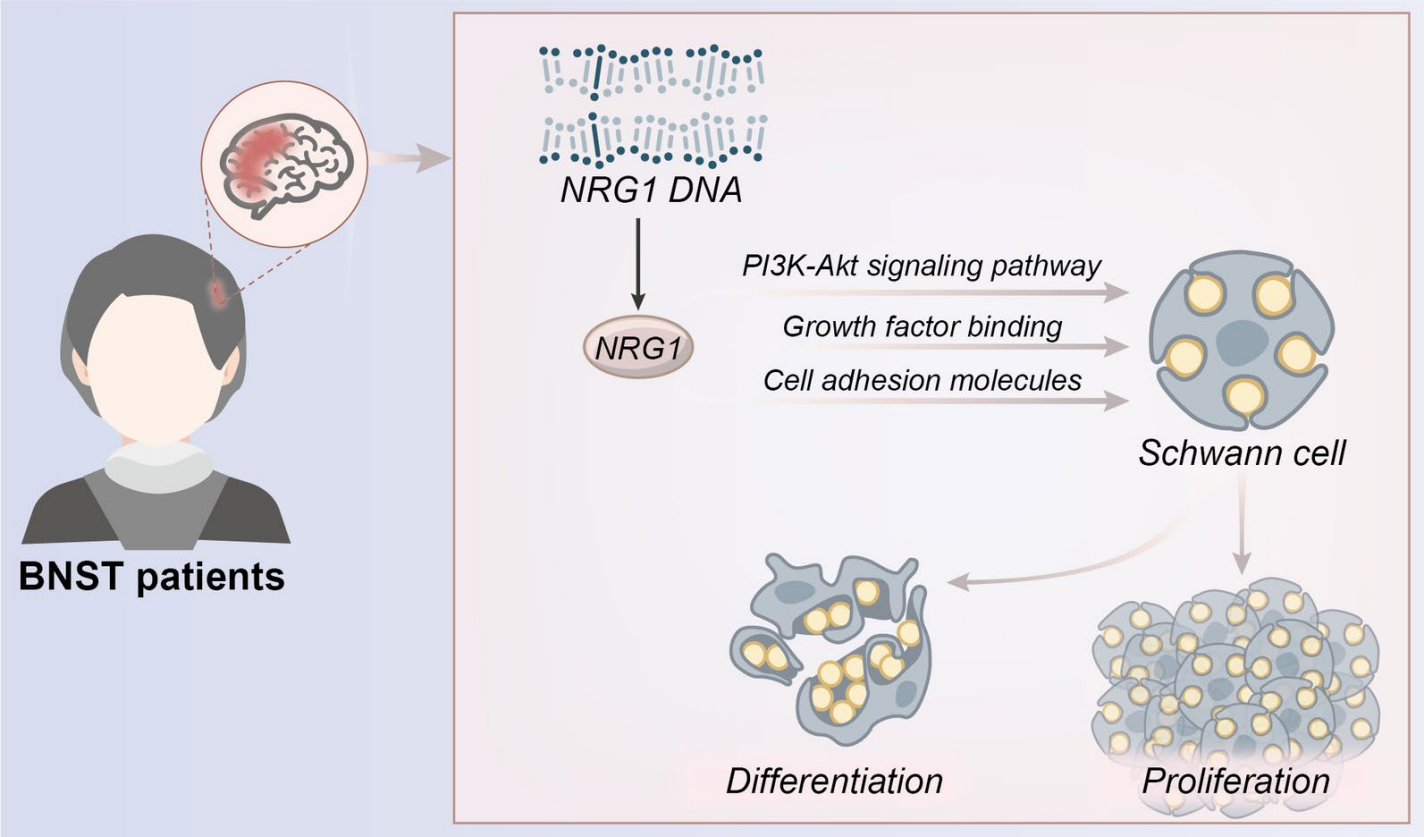
NRG1 affects tumor cell growth, proliferation, and survival through pathways such as PIK-Akt, participating in the occurrence and molecular mechanisms of BNST

 This study successfully uncovered the primary function of NRG1 in the development of the BNST (Fig. 11). In particular, NRG1 has been recognized as a highly promising diagnostic biomarker, offering unprecedented potential for the diagnosis and treatment of BNST. Nevertheless, each study possesses inherent limitations, such as the sample size, selected models, and specific methods employed. These limitations have the potential to impact both the generalizability and accuracy of the final conclusions. Based on these initial findings, it is necessary to further investigate the potential therapeutic uses of NRG1. Moreover, exploring additional pathways associated with NRG1 and investigating its relevance to other neurological disorders are crucial areas for future research. Such investigations have the potential to yield significant advancements in the fields of neurology and oncology.

### Supplementary Information

**Additional file 1:** **Table S1. **NRG1 knockdown sequence. **Table S2. **RT qPCR primer sequence.

**Additional file 2: Figure S1. **Morphological and pathological stability of the original tumor tissue and PDX xenografts. Note: (**A**) Workflow of clinical sample collection and PDX model construction. It displays the brain MRI of the patient, cells derived from the tumor, xenografts in the mouse femoral nerve (indicated by black arrows), and representative images of the cell line. SA-β-Gal staining was positive after 12 weeks of continuous passage; (**B**) Representative images of HE staining in the patient tumor and xenograft tumor, showing spindle cell lesions with mixed (Antoni A) and loose (Antoni B) cellular arrangements, Verocay bodies (indicated by blue arrows), and perivascular hyalinization (indicated by black arrows). Scale bar: 100 μm; (**C**) Representative images of S100, NRG1, and Ki67 immunohistochemical staining in the patient tumor and xenograft tumor. S100 is mainly expressed in BNST cytoplasm, NRG1 is mainly expressed in cytoplasm and membrane of Schwann cells and macrophages, and Ki67 is mainly expressed in the nuclei of proliferating cells. Scale bar: 100 μm; (**D**) Representative images of CD206 and CD66b immunohistochemical staining for M2 macrophages (M2 mac) and neutrophils (neu) in the patient tumor and xenograft tumor, respectively. CD206 and CD66b are expressed on the cell membrane of M2 macrophages and neutrophils, respectively. Scale bar: 100 μm; (**E**) Flow cytometry analysis of changes in M2 macrophages and neutrophils in the patient tumor and xenograft tumor (images sourced from [29]). M2 macrophages: CD68^+^CD206^+^, neutrophils: CD14^+^CD66b^+^. The graph on the right represents the distribution in the first quadrant; (**F**) Western blot analysis of control tissues, and changes in NRG1 and PI3K/AKT key proteins in patients and xenografts. “con” represents normal vestibular tissue. The graph on the right represents the protein expression levels; (**G**) Representative images of S100 and F-actin immunofluorescence staining in tumor-derived cells and cell lines. S100 (red) is distributed in the cytoplasm, while F-actin (green) is also distributed in the cytoplasm. DAPI (blue) is used to indicate the cell nuclei. Scale bar: 100 μm. *P*<0.05 compared to the “con” group, *P*<0.01 compared to the “con” group, *P*<0.001 compared to the “con” group, **P<0.0001 compared to the “con” group.

## Data Availability

The data that supports the findings of this study are available on request from the corresponding author upon reasonable request.
