# Supplementary figures and images for: Diagnostic potential of NRG1 in benign nerve sheath tumors and its influence on the PI3K-Akt signaling and tumor immunity
Source: Diagn Pathol. 2024 Feb 8;19:28. doi: 10.1186/s13000-024-01438-9 (PMC10851500; doi:10.1186/s13000-024-01438-9)

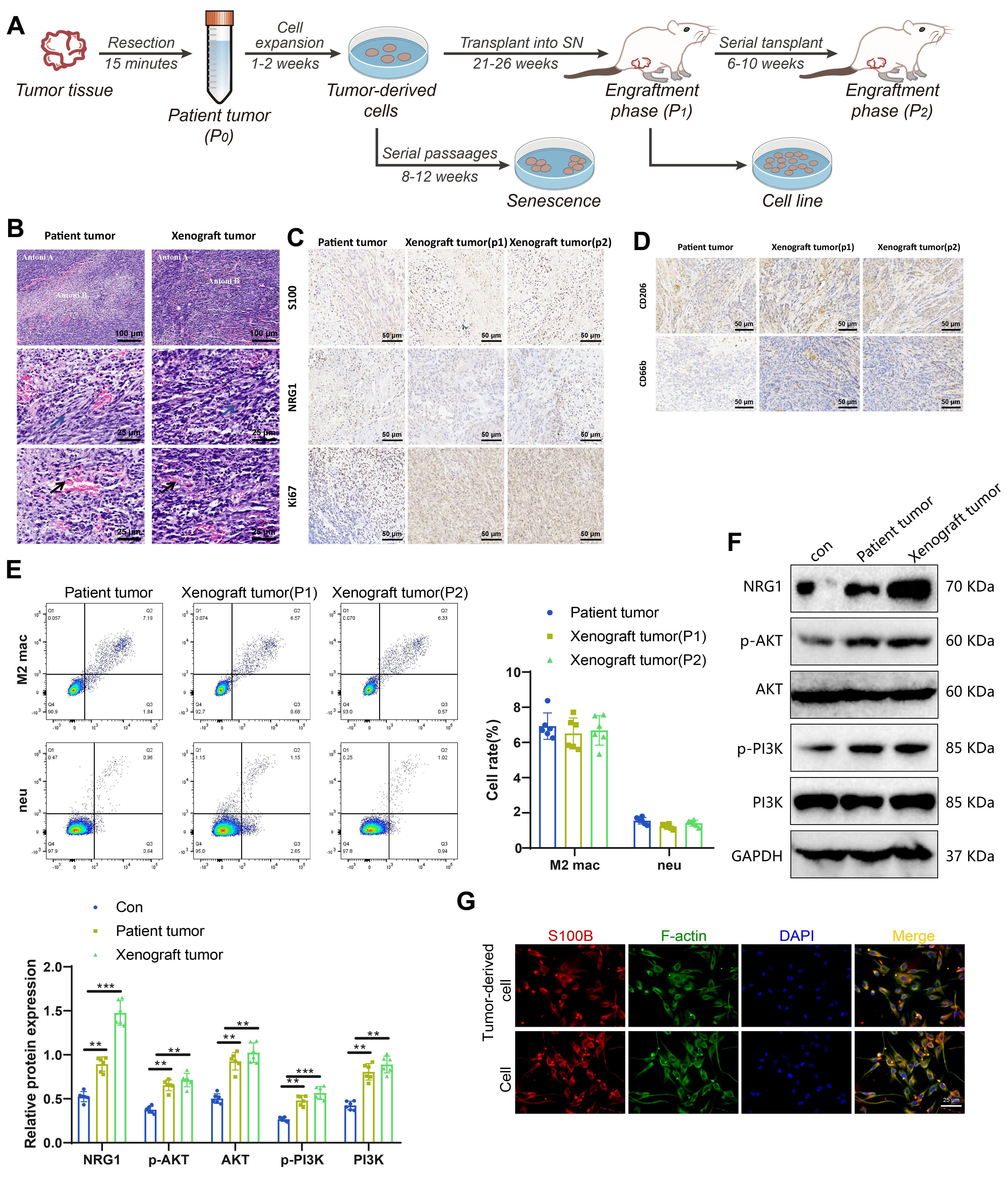

Supplement: Supplementary file 1 — Additional file 1: Table S1. NRG1 knockdown sequence. Table S2. RT qPCR primer sequence. [file 13000_2024_1438_MOESM1_ESM.jpg]
