# Supplementary material for: Diagnostic potential of NRG1 in benign nerve sheath tumors and its influence on the PI3K-Akt signaling and tumor immunity
Source: Diagn Pathol. 2024 Feb 8;19:28. doi: 10.1186/s13000-024-01438-9 (PMC10851500; doi:10.1186/s13000-024-01438-9)
Supplement: Supplementary file 2 — Additional file 2: Figure S1. Morphological and pathological stability of the original tumor tissue and PDX xenografts. Note: (A) Workflow of clinical sample collection and PDX model construction. It displays the brain MRI of the patient, cells derived from the tumor, xenografts in the mouse femoral nerve (indicated by black arrows), and representative images of the cell line. SA-β-Gal staining was positive after 12 weeks of continuous passage; (B) Representative images of HE staining in the patient tumor and xenograft tumor, showing spindle cell lesions with mixed (Antoni A) and loose (Antoni B) cellular arrangements, Verocay bodies (indicated by blue arrows), and perivascular hyalinization (indicated by black arrows). Scale bar: 100 μm; (C) Representative images of S100, NRG1, and Ki67 immunohistochemical staining in the patient tumor and xenograft tumor. S100 is mainly expressed in BNST cytoplasm, NRG1 is mainly expressed in cytoplasm and membrane of Schwann cells and macrophages, and Ki67 is mainly expressed in the nuclei of proliferating cells. Scale bar: 100 μm; (D) Representative images of CD206 and CD66b immunohistochemical staining for M2 macrophages (M2 mac) and neutrophils (neu) in the patient tumor and xenograft tumor, respectively. CD206 and CD66b are expressed on the cell membrane of M2 macrophages and neutrophils, respectively. Scale bar: 100 μm; (E) Flow cytometry analysis of changes in M2 macrophages and neutrophils in the patient tumor and xenograft tumor (images sourced from [29]). M2 macrophages: CD68+CD206+, neutrophils: CD14+CD66b+. The graph on the right represents the distribution in the first quadrant; (F) Western blot analysis of control tissues, and changes in NRG1 and PI3K/AKT key proteins in patients and xenografts. “con” represents normal vestibular tissue. The graph on the right represents the protein expression levels; (G) Representative images of S100 and F-actin immunofluorescence staining in tumor-derived cells and cell lines. S100 [file 13000_2024_1438_MOESM2_ESM.docx]

**Table S1.NRG1 knockdown sequence**

| Name | Knockdown sequence (5’-3’) |
| --- | --- |
| sh-NC | CCTAAGGTTAAGTCGCCCTCG |
| sh-NRG1 | GCAAGTGCCCAAATGAGTTTA |
| sh-NRG1-2 | GCTTCTACAGTACGTCCACTC |

The above sequence is composed of https://rnaidesigner.thermofisher.com/rnaiexpress/sort.do Website design and BLAST validation

**Table S2. RT qPCR primer sequence**

| Gene | primer sequence |
| --- | --- |
| NRG1 | Forward: 5’-ATGTGTCCCAAACCTGCTATCA-3’;  Reserve: 5’-GTGAGCCATGCGCCCTTTA-3’ |
| GAPDH | Forward: 5’- GGAGCGAGATCCCTCCAAAAT-3’;  Reserve: 5’- GGCTGTTGTCATACTTCTCATGG-3’ |
